# Acute depletion of the ARID1A subunit of SWI/SNF complexes reveals distinct pathways for activation and repression of transcription

**DOI:** 10.1016/j.celrep.2021.109943

**Published:** 2021-11-02

**Authors:** Seraina Blümli, Nicola Wiechens, Meng-Ying Wu, Vijender Singh, Marek Gierlinski, Gabriele Schweikert, Nick Gilbert, Catherine Naughton, Ramasubramanian Sundaramoorthy, Joby Varghese, Robert Gourlay, Renata Soares, David Clark, Tom Owen-Hughes

**Affiliations:** 1 Centre for Gene Regulation and Expression, School of Life Sciences, University of Dundee, Dundee DD1 5EH, UK; 2 Computational Core, University of Connecticut, 67 North Eagleville Road, Storrs, CT 06269, USA; 3 Computational Biology, School of Life Sciences, University of Dundee, Dundee DD1 5EH, UK; 4 MRC Human Genetics Unit, Institute of Genetics & Cancer, The University of Edinburgh, Edinburgh EH4 2XU, UK; 5 MRC Protein Phosphorylation and Ubiquitylation Unit, University of Dundee, Dundee DD1 5EH, UK; 6 Division of Developmental Biology, National Institute of Child Health and Human Development, NIH, Building 6A, 6 Centre Drive, Bethesda, MD 20892, USA

**Keywords:** ARID1A, SWI/SNF, EP300, BAF, chromatin remodeling, nucleosomes, enhancer transcription, cancer

## Abstract

The ARID1A subunit of SWI/SNF chromatin remodeling complexes is a potent tumor suppressor. Here, a degron is applied to detect rapid loss of chromatin accessibility at thousands of loci where ARID1A acts to generate accessible minidomains of nucleosomes. Loss of ARID1A also results in the redistribution of the coactivator EP300. Co-incident EP300 dissociation and lost chromatin accessibility at enhancer elements are highly enriched adjacent to rapidly downregulated genes. In contrast, sites of gained EP300 occupancy are linked to genes that are transcriptionally upregulated. These chromatin changes are associated with a small number of genes that are differentially expressed in the first hours following loss of ARID1A. Indirect or adaptive changes dominate the transcriptome following growth for days after loss of ARID1A and result in strong engagement with cancer pathways. The identification of this hierarchy suggests sites for intervention in ARID1A-driven diseases.

## Highlights

• Degradation of ARID1A disrupts nucleosomes flanking pluripotency transcription factors
• EP300 is rapidly redistributed with increased occupancy adjacent to upregulated genes
• These changes are associated with misregulation of a few hundred genes within 2 h
• During subsequent days, widespread indirect changes mimic a premalignant state

## Graphical abstract

**Figure:**
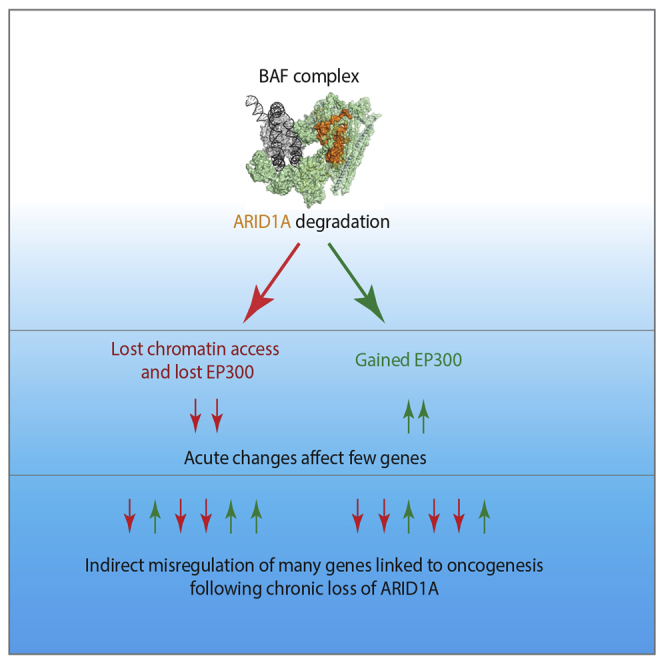

## Teaser

Blümli et al. show that degradation of ARID1A reorganizes nucleosomes flanking pluripotency transcription factors and causes redistribution of the coactivator EP300. Lost EP300 at enhancers is associated with downregulation of transcription and gained EP300 with upregulation. Few genes are directly affected, but widespread indirect effects accumulate slowly to phenocopy premalignancy.

## Introduction

One of the ways by which eukaryotes regulate chromatin structure is through the action of ATP-dependent chromatin remodeling activities (Narlikar et al., 2013). The first ATP-dependent chromatin remodeling complex to be characterized was the budding yeast SWI/SNF complex. The catalytic ATPase within the yeast SWI/SNF complex, the *SNF2* or *SWI2* gene, is conserved through to humans (Kadoch and Crabtree, 2015) and is typically associated as a series of complexes with some 10–20 accessory subunits. Many but not all of these accessory subunits are also conserved, and biochemically these complexes reconfigure nucleosomes (Clapier et al., 2017). In mammals there are three main forms of the complex with distinct subunit compositions (Mashtalir et al., 2018).

Mutations to these complexes have long been recognized as causing alterations to the transcription of a subset of genes. Deletion of the yeast Snf2 protein results in both upregulation and downregulation of hundreds of genes (Holstege et al., 1998; Sudarsanam et al., 2000), but modest effects on global chromatin organization (Ganguli et al., 2014). In contrast, depletion of the Snf2 paralog Sth1 results in rapid organization of chromatin at nucleosome-free promoter regions (Ganguli et al., 2014; Klein-Brill et al., 2019). This is associated with a shift in the position of the +1 nucleosome toward promoters affecting adjacent coding region nucleosomes but relatively minor primary defects to transcription (Klein-Brill et al., 2019).

In multicellular eukaryotes from *Drosophila* to humans, it has similarly been observed that loss of function to subunits of SWI/SNF complexes results in both upregulation and downregulation of transcription (Ho et al., 2011; Hodges et al., 2018; Langer et al., 2019; Moshkin et al., 2007; Tolstorukov et al., 2013). Loss of function of a subunit is also linked with substantial changes to chromatin accessibility predominantly at enhancer elements that are also associated with loss of histone modifications, including H3K27 acetylation and H3K27 methylation (Alver et al., 2017; Barisic et al., 2019; Ho et al., 2011; Hodges et al., 2018; Langer et al., 2019; Rada-Iglesias et al., 2011; Wang et al., 2017; Zhang et al., 2014).

Determining the pathways through which SWI/SNF complexes act requires the analysis of time series data. There are a number of examples of such data that indicate different relationships between ATP-dependent chromatin structure and other changes to chromatin, including histone modifications and transcription at the interferon (IFN)-β locus and PS2 promoter (Agalioti et al., 2000; Métivier et al., 2003). More recently, directed recruitment of SWI/SNF complexes to an engineered *Pou5f1* allele shows that this results in sequential removal of PRC1 complexes, histone H2A ubiquitylation, PRC2 complexes, and histone H3K27 methylation, ultimately increasing chromatin accessibility (Kadoch et al., 2017). A caveat to all of these studies is that they involve the establishment of a temporal pathway at individual loci making it difficult to assess how general the findings are. An analysis of the global changes to chromatin that explains both the upregulation and downregulation of gene expression following loss of mammalian SWI/SNF complexes has remained elusive.

Following the advent of population-based genome sequencing, it has emerged that multiple subunits of human forms of the SWI/SNF complex are mutated at high rates in tumors. For example, the PBRM1 subunit is mutated in 40% clear cell renal cancer. In contrast, the ARID1A subunit is mutated in approximately 50% of ovarian clear cell carcinoma, 30% of endometrial cancer (Jones et al., 2010; Wiegand et al., 2010), 25% of gastric cancer (Wang et al., 2011), and 25% of bladder cancer (Gui et al., 2011). These mutations are predominantly truncating, and in the case of ovarian tumors, patient tissue is depleted for ARID1A protein (Wiegand et al., 2010). While ARID1A null mice are inviable (Gao et al., 2008), conditional knockout of ARID1A in concert with activating mutations of PIK3CA promotes growth of tumors resembling ovarian clear cell carcinoma (Chandler et al., 2015; Wilson et al., 2019). As a result of ARID1A’s function as a tumor suppressor, it is of special interest to determine how the function of SWI/SNF complexes is perturbed following loss of this subunit.

ARID1A is a subunit of the BAF form of mammalian SWI/SNF complexes (Mashtalir et al., 2018; Sif et al., 2001; Wang et al., 1996) where its presence is mutually exclusive with the paralog ARID1B (Raab et al., 2015). Loss of ARID1A has been shown to result in changes to chromatin accessibility and histone modifications, including H3K4me1, H3K27ac, and H3K27me3 at enhancers (Kelso et al., 2017; Mathur et al., 2017), and both increases and decreases in transcription at a subset of genes (Kelso et al., 2017; Mathur et al., 2017), including those driving epithelia invasion (Wilson et al., 2019). However, the temporal relationship between these changes is unclear.

Here we utilize an auxin degron system (Natsume et al., 2016) to investigate the response to degradation of ARID1A over time and gain insight into its mechanism of action. We identify rapid changes to chromatin at thousands of sites. The co-activator EP300 is also rapidly redistributed following ARID1A loss. The relatively small subset of locations where chromatin accessibility and EP300 association are reduced occur adjacent to downregulated genes. Following ARID1A degradation, EP300 associates and drives rapid but indirect transcriptional activation. These distinct activating and repressive pathways initiate changes to transcription at a small number of genes that propagate additional indirect changes over the following days during which cells converge to a state resembling that of an ARID1A^−/−^ null cell line.

## Results

### Engineering mouse ESCs for rapid depletion of ARID1A from SWI/SNF complexes

**Figure 1:**
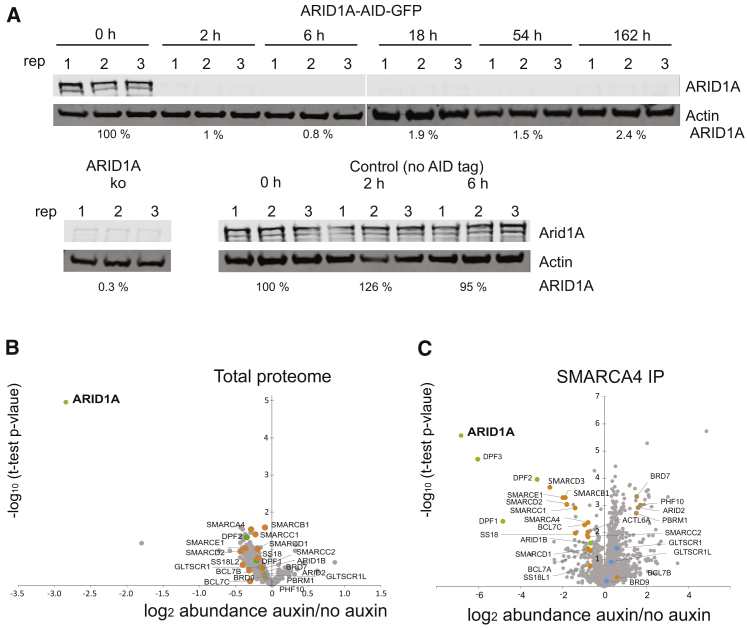
Establishment of a degron to specifically degrade the ARID1A subunit of SWI/SNF complexes (A) Western blot showing ARID1A depletion in mouse ESCs. mESCs expressing endogenous ARID1A protein homozygously tagged with mAID-GFP after the addition of 500 μM auxin for indicated time points, and ARID1A abundance in cells lines where ARID1A is knocked out or not tagged. (B) The abundance of total cell proteins prior to and 2 h following growth in the presence of auxin indicates that ARID1A is specifically targeted for degradation via the degron. (C) The abundance of SMARCA4-associated proteins determined by mass spectrometry is indicated as fold change of protein abundance prior to and 2 h following addition of auxin to cell media. Other than BAF-specific subunits ARID1A, DPF1, and DPF2 other subunits remain substantially associated with SMARCA4.

In order to define direct targets affected by loss of function of mammalian SWI/SNF complexes, a degron strategy was adopted. The endogenous ARID1A gene was homozygously tagged with the mini-auxin-induced degron (mAID) targeting peptide and GFP (Natsume et al., 2016) in a mouse embryonic stem cell (ESC). Tagged ARID1A is expressed at levels comparable to its levels in untagged cells (Figure 1A). In the presence of auxin, TIR1 directs ubiquitylation and subsequent proteolysis of proteins fused to the mAID peptide (Natsume et al., 2016). Consistent with this, western blotting following addition of auxin indicates that ARID1A is substantially degraded between 20 min and 2 h and that degradation can be maintained in culture for more than 162 h (Figure 1A). Tandem mass tag (TMT) whole-cell proteomics after 2 h of auxin treatment indicates that ARID1A is the major target for auxin-dependent degradation following growth in the presence of auxin (Figure 1B).

SWI/SNF complexes immunopurified before and 2 h following auxin addition exhibit strong depletion of ARID1A (Figure 1C). DPF1–DPF3 proteins, also known as BAF45B, BAF45D, and BAF45C, are substantially depleted following loss of ARID1A. Several subunits contributing to the core of the BAF complex (He et al., 2020), including SMARCD3, SMARCE1, SMARCD2, SMARCC1, and SMARCB1 are partially dissociated from SMARCA4, consistent with previously observed destabilization of these subunits following loss of ARID1A and ARID1B (Helming et al., 2014; Mashtalir et al., 2018). The depletion rather than complete dissociation of these subunits is partial, consistent with their association with SMARCA4 in the absence of ARID1A (He et al., 2020).

### ARID1A degradation triggers widespread changes to chromatin accessibility over different timescales

**Figure 2:**
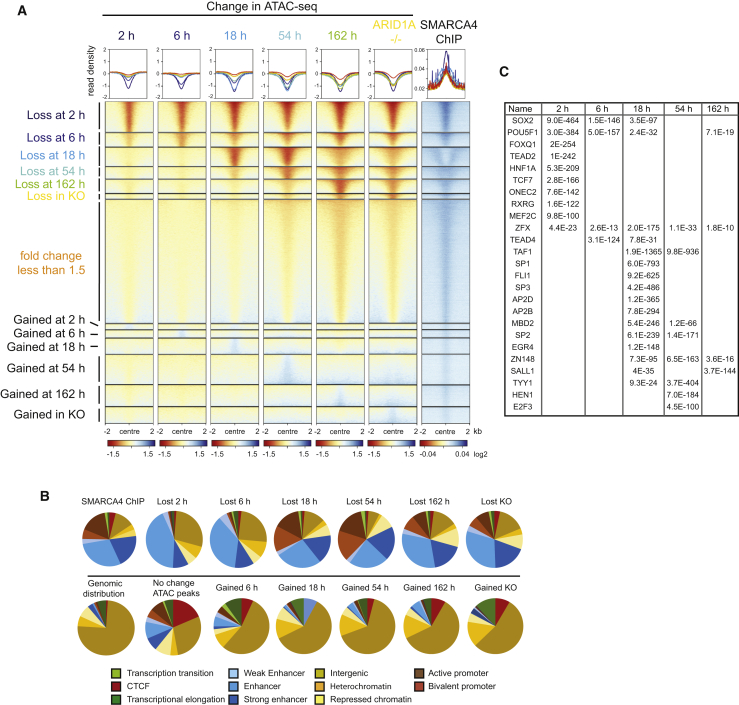
Chromatin changes at different sites over time following ARID1A degradation (A) Density plots of lost (red) and gained (blue) ATAC-seq signals changing at time points (2, 6, 18, 54, and 162 h) following degradation of ARID1A and in knockout (ARID1A^−/−^) mESCs. The list of sites is ordered by the time at which a change of >1.5-fold is first detected. (Last panel) Enrichment for ChIP-seq signal of the ATPase SMARCA4 (de Dieuleveult et al., 2016) at the same ATAC sites. (B) Distribution of SMARCA4 ChIP-seq peaks and lost ATAC changes across ESC chromatin states (Pintacuda et al., 2017). Color code for chromatin states defined by histone modifications and transcription factor binding below the panel. (C) Motif-based sequence analysis of ATAC peaks lost after 2, 6, 18, 54, and 162 h of ARID1A depletion. E values given show the significance of the motif as reported by MEME-ChIP (Motif Analysis of Large Nucleotide Datasets) (Machanick and Bailey, 2011). Only factors with an E value of 1 × 10 to 1 × 100 or lower are included.

In order to assess the functional consequences of ARID1A depletion, changes to chromatin accessibility were monitored using ATAC-seq (assay for transposase accessible chromatin with high-throughput sequencing) (Corces et al., 2017). After 2 h, chromatin changes were detected at 11,488 of the 39,876 sites called in untreated cells (Figure 2A, loss at 2 h). Additional, smaller cohorts of sites lose chromatin accessibility at successive time points (Figure 2A). Chromatin changes after 162 h are most similar to those of an ARID1A knockout, indicating that prolonged auxin treatment recapitulates chromatin changes detected following chronic loss of function.

In addition to sites of lost chromatin accessibility, sites at which chromatin accessibility is increased are also detected during the time course. In general, these sites are detected later in the time series and are transient (Figure 2A). The localization of the SMARCA4 subunit of SWI/SNF complexes shows some enrichment at all sites of accessible chromatin, including those not affected by loss of ARID1A (Figure 2A). However, association is strongest at sites where chromatin accessibility is lost rapidly in comparison to sites of gained or slowly lost accessibility (Figure 2A). This is consistent with rapidly lost accessibility occurring directly as a result of ARID1A loss, while the other changes are likely to be indirect.

### Acute ARID1A-dependent chromatin accessibility changes occur within enhancer chromatin

ARID1A-dependent changes to chromatin accessibility were mapped onto a chromatin state model for mouse ESC chromatin (Pintacuda et al., 2017). Changes occurring after growth in the presence of auxin for 2 and 6 h were strongly enriched in enhancer chromatin types, and distinct from the distribution of ATAC peaks that do not change following ARID1A depletion (Figure 2B). The early changes are detected in all three enhancer classes, but of these, the “enhancer” is predominant. Sites of lost chromatin accessibility occurring 18 and 54 h following ARID1A degradation are observed at both enhancers and promoters while at later time points the prevalence at enhancers is restored (Figure 2B). Sites of gained chromatin accessibility are distributed across chromatin states, raising the possibility that these changes are not specifically targeted (Figure 2B).

### Binding sites for pluripotency transcription factors are enriched in accessible chromatin acutely sensitive to ARID1A loss

To investigate interplay between the function of SWI/SNF complexes and sequence-specific transcription factors, the enrichment of DNA binding motifs at sites of ARID1A action was determined. Motifs related to pluripotency transcription factors POU5F1 (OCT3/4), SOX2, the winged helix factor FOXQ1, homeobox transcription factors HNF1A, and nuclear receptor RXRG that all contribute to the transcriptional landscape of ESCs are all highly enriched at sites where chromatin accessibility is lost after 2 h (Figure 2C). Interestingly, sites that lose chromatin accessibility at subsequent time points were enriched for the binding sites of distinct cohorts of transcription factors. For example, sites losing accessibility after 18 h show very high enrichment for the binding sites of promoter-proximal transcription factors and TAF1. This is consistent with the enrichment for changes in promoter chromatin at these time points (Figure 2B). These changes are likely to be indirect, as chromatin immunoprecipitation (ChIP) for SMARCA4 is reduced at sites where chromatin changes occur at later time points.

### BAF complexes organize clusters of nucleosomes at the binding sites of pluripotency transcription factors

**Figure 3:**
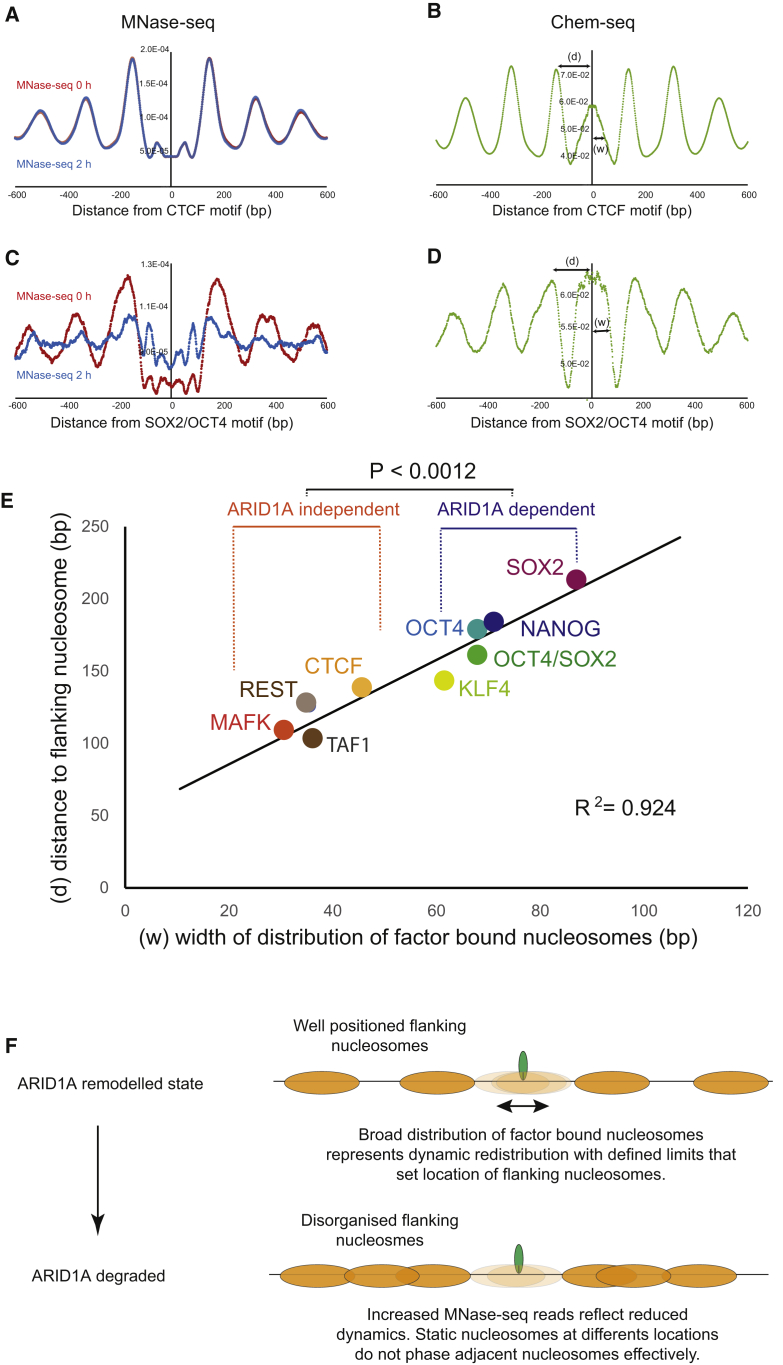
ARID1A organizes clusters of nucleosomes adjacent to the binding sites of pluripotency transcription factors (A) The centers of nucleosomal fragments protected from MNase digestion obtained prior to (red) and 2 h following degradation of ARID1A (blue) are shown aligned to the consensus binding site for CTCF. (B) The distribution of chemical cleavage directed from histone H4 S47C (Voong et al., 2016) aligned to CTCF sites is shown in green. The distance to the adjacent nucleosome (d) and the width of the distribution of histone contacts (w) are indicated. (C and D) Distributions of the same datasets at combined SOX2/OCT4 consensus motifs. (E) Plot of the distance (d) between adjacent nucleosomes and the width of the distribution (w) at the DNA binding motifs for a range of transcription factors (see Figure S2). (F) The limits of the distribution of ARID1A-sensitive transcription factor-bound nucleosomes have properties expected of a barrier capable of setting the locations of adjacent nucleosomes. In the absence of ARID1A a static distribution of nucleosomes across the factor binding site causes adjacent nucleosomes to be positioned heterogeneously.

Nucleosomes are precisely positioned adjacent to the binding sites of a subset of transcription factors (Wang et al., 2012). To characterize how nucleosome organization is affected by loss of ARID1A, MNase-seq (micrococcal nuclease sequencing) was performed at the 0- and 2-h time points. Well-organized arrays of nucleosomes are evident adjacent to the binding sites for CTCF, but these are little changed following loss of ARID1A (Figure 3A). Similarly, changes to nucleosome organization are not observed at the binding sites for REST, TAF1, or MAFK (Figure S1). However, at the binding sites for SOX2/OCT4, changes to nucleosome organization are observed with an increase in reads recovered in the region spanning the factor binding site and a reduction in the positioning of flanking nucleosomes (Figure 3C). Similarly, ARID1A is observed to organize nucleosomes adjacent to binding sites for OCT4, KLF4, and NANOG (Figure S1).

To gain further insight into the nature of nucleosomes configured through the action of ARID1A-containing complexes, sites of DNA cleavage directed by chemistry coupled to histone H4 were interrogated (Voong et al., 2016). These data have previously been used to show that nucleosomes are present at many sites bound by transcription factors (Voong et al., 2016). Alignment of Chem-seq data to SOX2/OCT4 sites shows that histone-DNA contacts are similar in magnitude to those observed within the flanking nucleosomes and are broadly distributed, spanning approximately 70 bp on either side of the binding sites (Figure 3D). In contrast, the distribution of chemical cleavages is relatively weak and tightly distributed with approximately 35 bp on either side of transcription factors such as CTCF, where nucleosome organization is independent of ARID1A (Figure 3B). This trend for a broader distribution of nucleosomes at the binding sites for ARID1A-dependent factors holds true across additional transcription factor binding sites (Figure S1). Centrally located nucleosomes are tightly distributed at the binding sites of ARID1A-independent factors such as CTCF, REST, and MAFK but distributed broadly at SOX2, NANOG, KLF4, and OCT4. Moreover, adjacent nucleosomes are located farther away from binding motifs, where nucleosomes are broadly distributed (Figure 3E). In combination, these findings indicate that ARID1A-containing complexes act to generate a broader distribution of nucleosomes across the binding sites of selected transcription factors and that this in turn has the properties expected of a barrier capable of setting the location of adjacent nucleosomes. In this way, ARID1A-containing complexes organize clusters of three to seven nucleosomes that comprise an accessible minidomain (Figure 3F).

### Changes to the transcriptional landscape following loss of ARID1A

**Figure 4:**
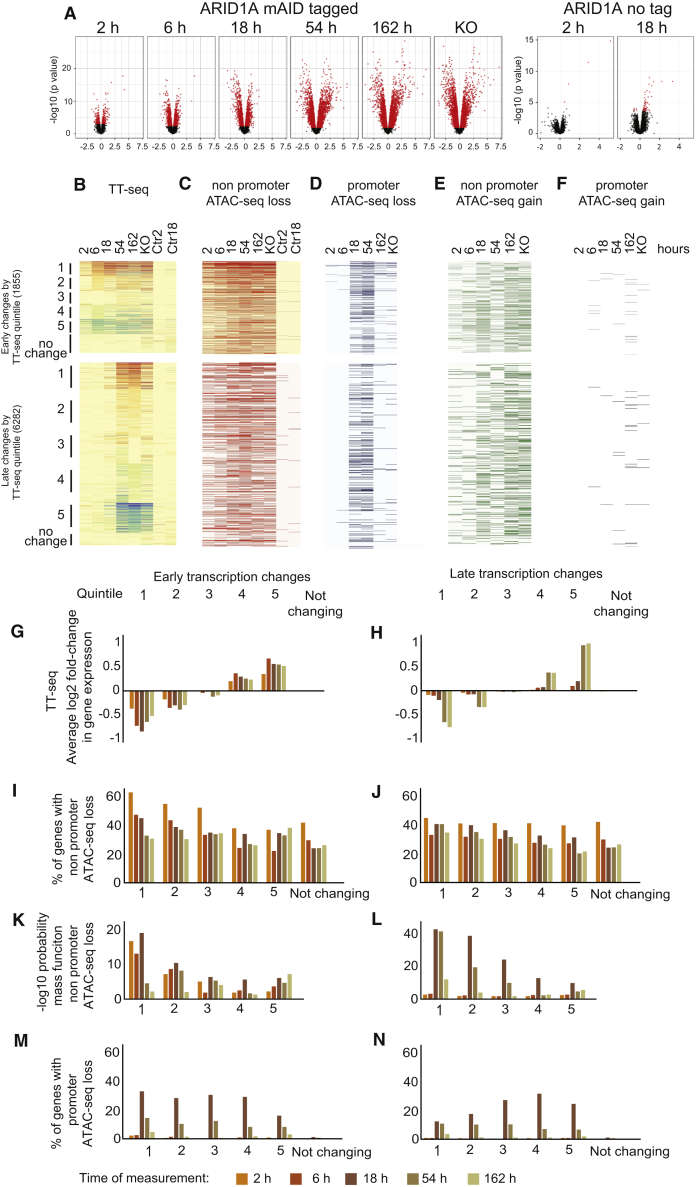
Loss of enhancer chromatin accessibility is enriched at downregulated genes (A) The numbers of genes changing significantly (red) at each time point represented as volcano plots. Right panels indicate changes following incubation of mESCs that do not have ARID1A mAID tagged with auxin. (B) Transcription for genes changing (FDR < 0.05) after 2 and 6 h following ARID1A degradation and those changing later is plotted for groups sorted into quintiles based on the fold change at 6 and 162 h. A group of 500 genes selected based on no change to transcription following depletion of ARID1A is included for comparison. Changes in TT-seq at each time point are represented as a heatmap in (B). Also shown are the changes in an ARID1A −/− line and control lines in which ARID1A is not degron tagged. The average log_2_ fold change in expression for genes in each quintile at each time point is plotted as a histogram for early changing genes in (G) and for late genes in (H). (C) Loss of ATAC-seq (> 1.5-fold) in the 50 kb region (excluding promoters) adjacent to differentially expressed genes. (D) Loss of ATAC-seq (> 1.5-fold) at promoters (−500 to +500 bp from the TSS). (E) Gain of ATAC-seq in regions 50 kb either side of the promoters of each TT-seq gene. (F) Gained promoter ATAC-seq. (G and H) Quintiles represent: 1, top 20% of repressed genes; 2, top 20%–40% of repressed genes; 3, top 40%–60% of genes; 4, top 20%–40% of activated genes; 5, top 20% of activated genes. (I and J) Bar graphs showing the percentage of genes in each differentially expressed group of genes that have loss of chromatin accessibility within 50 kb on either side of the TSS. The different colored bars represent the percentage of genes that have sites of lost chromatin accessibility first detected at successive time points according to the key. (K and L) −Log_10_ probability mass function for the enrichments in (I) and (J) are plotted indicating the likelihood that the enrichments would occur by chance. (M and N) Percentages of genes in each group with promoter chromatin changes. In each histogram bars are colored according to the time at which changes in ATAC-seq are detected as indicated in the key.

Changes to nascent transcription following loss of ARID1A were monitored by TT-seq (transient transcriptome sequencing) (Schwalb et al., 2016). Initially, relatively few genes are affected; however, increasing numbers of genes are both upregulated and downregulated at later time points (Figure 4A). Importantly, changes after growth in the presence of auxin for 162 h correlate well with those in an ARID1A^−/−^ line, indicating that prolonged auxin depletion recapitulates transcriptional changes observed in a knockout line (Figure S2A). This indicates that the transcriptional landscape develops from effects at few genes at early time points to widespread changes after several days. Furthermore, the large numbers of genes differentially expressed late in the time series show modest changes at early time points (Figures 4A and S2B).

A small cohort of four genes are differentially expressed (false discovery rate [FDR] < 0.05) following culture of a cell line in which TIR1 is expressed, but ARID1A is not tagged in the presence of auxin for 2 h (Figure 4A). These include the CYP1B1 and CYP1A1 genes known to be regulated to the aryl hydrocarbon receptor (Zanger and Schwab, 2013), which is activated following exposure to polycyclic aromatic hydrocarbons, including auxin (Sathyan et al., 2019). Otherwise, the transcriptional response exhibits high specificity for both auxin and mAID tagging of ARID1A.

### Loss of chromatin accessibility at enhancer sites occurs adjacent to downregulated genes

To identify cohorts of genes that are co-regulated, the genes that show significant (FDR < 0.05) changes in transcription prior to (early) and after (late) 6 h were separately ranked as quintiles based on differential expression. 1,855 genes meet the FDR threshold at the early time points while 6,282 genes are differentially expressed at later time points (Figures 4B, 4G, and 4H). 4,603 genes are not differentially expressed (FDR < 0.05) at any time point, and 500 of these are included as a control grouping of non-differentially expressed genes.

To investigate the relationship between transcriptional changes and chromatin changes, the percentage of genes with at least one chromatin change within each quintile was calculated. Promoter changes were assigned as changes within 500 bp of the transcription start site (TSS); enhancer changes occur in a region extending to 50 kb either side of the promoter region. Loss of chromatin accessibility is observed at many sites that are not obviously correlated with transcriptional changes. For example, sites of chromatin accessibility lost after 2 h are detected adjacent to 40% of genes that are not differentially expressed (Figures 4D–4I). However, rapid loss of chromatin accessibility is detected adjacent to 65% of rapidly downregulated genes (Figures 4C and 4I). This enrichment adjacent to downregulated genes is very unlikely to occur by chance, as indicated by the low probability mass function (Figure 4K). Early changes to chromatin accessibility are not enriched at sites adjacent to genes differentially regulated later in the time series (Figure 4J). However, there is a small increase in the number of genes with sites of lost chromatin accessibility detected at later time points, in comparison to not changing or upregulated genes (Figure 4J). These may represent indirect or adaptive changes to chromatin that are associated with downregulation of gene expression at later time points.

### Changes to promoter chromatin accessibility are weakly correlated with transcriptional changes

Approximately one third of the sites of lost chromatin accessibility first detected 18 and 54 h following ARID1A degradation are located at active and bivalent promoters (Figure 2B) and are enriched in binding sites for promoter-proximal transcription factors, including TAF1 (Figure 2C). Loss of promoter chromatin accessibility at these time points is distributed across upregulated and downregulated genes (Figures 4D, 4M, and 4N) and so is unlikely to cause transcriptional changes.

### Sites of coincident loss of EP300 and chromatin accessibility are strongly coupled with transcriptional downregulation

The findings presented above indicate that loss of chromatin accessibility occurs at many thousands of sites following loss of ARID1A, but only a small proportion of these are linked to genes that are differentially transcribed. This raises the question, what distinguishes functional and non-functional chromatin changes?

To identify ARID1A interacting factors, ARID1A complexes were immunopurified followed by mass spectrometry. In addition to the expected SWI/SNF components, EP300 was observed to associate with ARID1A (Figure S3A) (Alver et al., 2017). Reciprocal immunoprecipitations (IPs) with EP300 resulted in enrichment of SWI/SNF components, including ARID1A (Figure S3B). Following induction of the auxin degron this association is reduced (Figure S3C), indicating that association of EP300 with SWI/SNF complexes is dependent on the ARID1A subunit.

**Figure 5:**
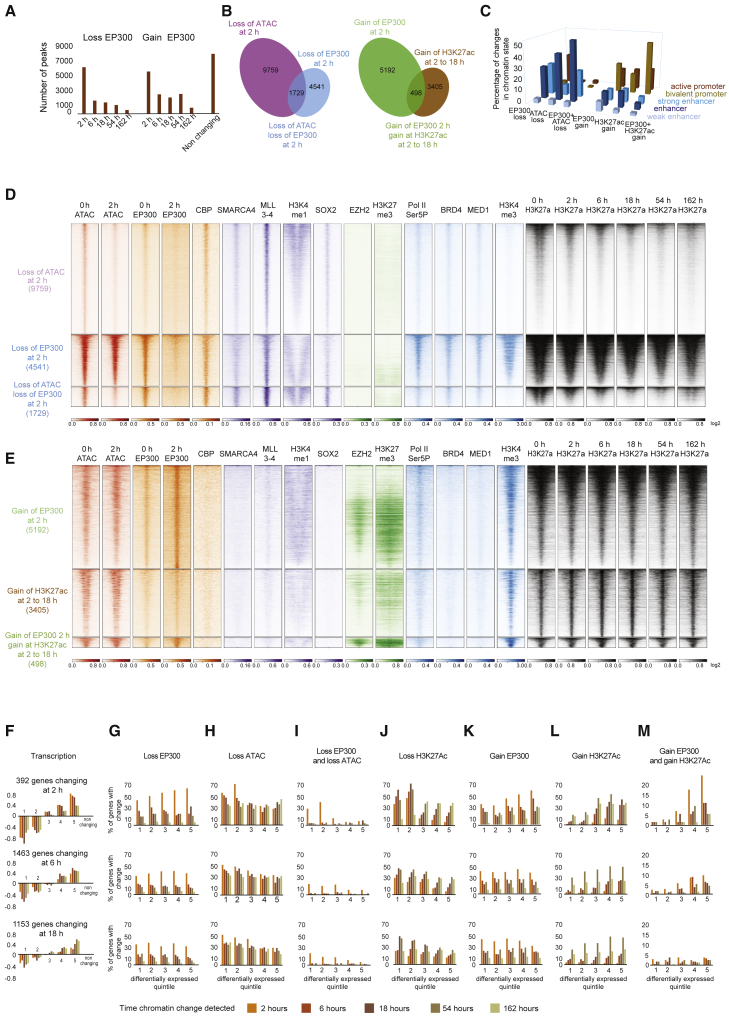
ARID1A-dependent EP300 occupancy in different chromatin contexts is associated with upregulation and downregulation of gene expression (A) The number of sites at which EP300 occupancy (FDR < 0.2 and fold change > 1.2) changes are detected are shown for each time point following degradation of ARID1A. (B) Intersects between called changes in ATAC-seq, EP300 occupancy and histone acetylation. (C) The distribution of the chromatin changes indicated in (B) across promoter and enhancer chromatin states (Pintacuda et al., 2017). (D and E) Heatmaps showing enrichment for selected factors at the genes where chromatin changes indicated in (B) occur. Heatmaps are centered with 2 kb on either side. (F) Average changes in transcription detected by TT-seq for quintiles of genes sorted by fold-change in expression and changing (FDR < 0.05) at 2, 6 and 18 h. (G–M) The percentage of differentially expressed genes shown in (F) that have the indicated chromatin changes within a 50-kb flanking region that excludes the promoter (G–J). Sites of gained EP300 and histone acetylation are promoter proximal, so the proportion of genes with these changes is calculated including promoters (K–M). Data relating to the genes differentially expressed at 2, 6, and 18 h are shown in the top middle and bottom row of histograms. Each histogram is organized as a block of data for each differentially expressed quintile of genes that are first differentially expressed at the time point. For each quintile a different colored bar indicates the chromatin changes first detected at that time point. (M) Sites of histone acetylation accumulated during 2–18 h that coincide with gained EP300 occupancy at each time point.

ChIP-seq was performed to survey EP300 occupancy following ARID1A degradation. EP300 is retained at some 8,000 sites following degradation of ARID1A but dissociates from some locations, with the greatest changes in occupancy observed after 2 h (Figure 5A). EP300 is lost from 4,541 locations 2 h following loss of ARID1A in the absence of chromatin changes (Figure 5D). Chromatin accessibility is lost at 9,759 locations in the absence of called changes in EP300 occupancy (FDR < 0.2 and fold change of 1.2); however, at these locations there is some loss of EP300 below this threshold (Figure 5D). The strongest changes to chromatin accessibility and EP300 occupancy are coincident and are detected at just 1,729 locations that are strongly enriched at enhancer chromatin types (Figure 5C).

**Figure 6:**
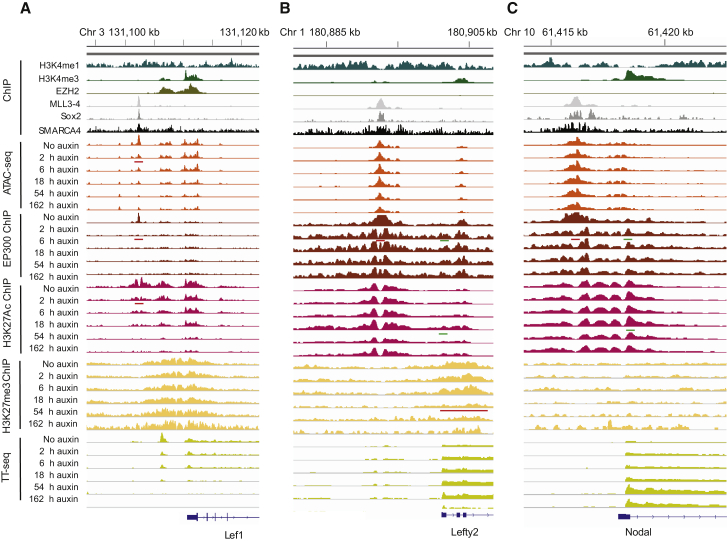
Changes to chromatin and transcription following ARID1A degradation at the Lef1 and Lefty2 loci (A–C) Distribution of selected factors at the Lef1 (A), Lefty2 (B), and Nodal (C) loci. Changes to chromatin accessibility (ATAC-seq), EP300, histone H3K27 acetylation, histone H3K27 methylation, and nascent transcription (TT-seq) are shown at the indicated times following addition of auxin. Sites of lost and gained occupancy are indicated by red and green lines.

To investigate the interplay between these changes and transcription, genes were scored based on whether each class of alteration occurred within 50 kb. Cohorts of co-regulated genes were sorted based on the time at which they first undergo a significant (FDR < 0.05) change in expression and then into quintiles based on the fold change in transcription (Figure 5F). Sites of reduced EP300 occupancy after 2 h are observed adjacent to many genes but are detected at similar frequency adjacent to those that are upregulated or downregulated (Figure 5G). In contrast, sites where chromatin accessibility is lost are enriched adjacent to downregulated genes (Figure 5H). The small number of sites where both ATAC signal and EP300 are lost are very highly enriched adjacent to downregulated genes and seldom found flanking upregulated genes (Figure 5I). Probability mass functions for these enrichments indicate that they are very unlikely to occur by chance (Figure S4). Sites of dual loss of chromatin accessibility have the highest enrichment for SMARCA4, MLL3 and MLL4 complexes, which function interdependently with BAF complexes (Park et al., 2021), and pluripotency transcription factors such as SOX2 (Figure 5D). Histone H3K27 acetylation is initially high at these sites and decreases over time following loss of ARID1A (Figure 5D). The loss of histone acetylation is delayed with respect to transcriptional downregulation. For example, loss of histone is maximal after 18 h at genes downregulated after 2 h (Figure 5J). Coincident loss of EP300 and ATAC peaks is prominent adjacent to individual downregulated genes such as Lef1 (Figure 6A).

A relatively small subset of the locations where EP300 is lost but chromatin accessibility does not change are associated with upregulated genes and may be a subset of sites at which EP300 functions as a co-repressor (Girdwood et al., 2003) (Figure S4J).

### Sites of ARID1A-dependent gain of EP300 occupancy and sites of increased histone acetylation are linked to transcriptional upregulation

Following ARID1A degradation, EP300 dissociates from 5,785 locations, but it associates at other sites (Figure 5A). This is consistent with increased association at locations that were previously partially occupied. Sites of gained EP300 occupancy are associated with about 50% of genes that are rapidly upregulated (Figure 5K). Increased H3K27 acetylation is also enriched 2–54 h following degradation of ARID1A in the regions adjacent to upregulated genes (Figure 5L). 14% of locations where acetylation increases are called overlap with regions that EP300 is gained. This is likely to be an underestimate of coincident changes due to the difficulty in correctly calling smaller changes in EP300 occupancy. Dual gained EP300 and acetylation sites are more strongly enriched adjacent to upregulated genes than sites where either change is called alone (Figure 5M). All three combinations share the properties that they are associated with minimal changes to chromatin accessibility, have initially very low ChIP for SMARCA4, have relatively high H3K4 trimethylation (Figure 5E), and are observed in promoter chromatin types (Figure 5C).

Some of these promoter-proximal sites are associated with high H3K27 methylation and PRC2 components, suggesting they represent bivalent promoters (Figure 5E). Changes to H3K27 methylation were monitored over time following depletion of ARID1A, and modest and relatively slow reduction of H3K27 methylation is observed at promoter-proximal sites (Figure S5). At individual loci these changes are observed to occur in different combinations. For example, at Lefty2 EP300 occupancy is gained and initially high H3K27 methylation reduces over time while at the upregulated Nodal gene, EP300 occupancy also increases at a promoter-associated site, but the locus is not associated with high initial K27 methylation (Figure 6). Consistent with this, sites of dual gained EP300 and histone acetylation are observed at both active and bivalent promoters (Figure 5C), and more of the upregulated genes fit the Bernstein definition of H3K4 methylated than bivalent (Mikkelsen et al., 2007).

### Progressive engagement with cancer and pluripotency signaling pathways

**Figure 7:**
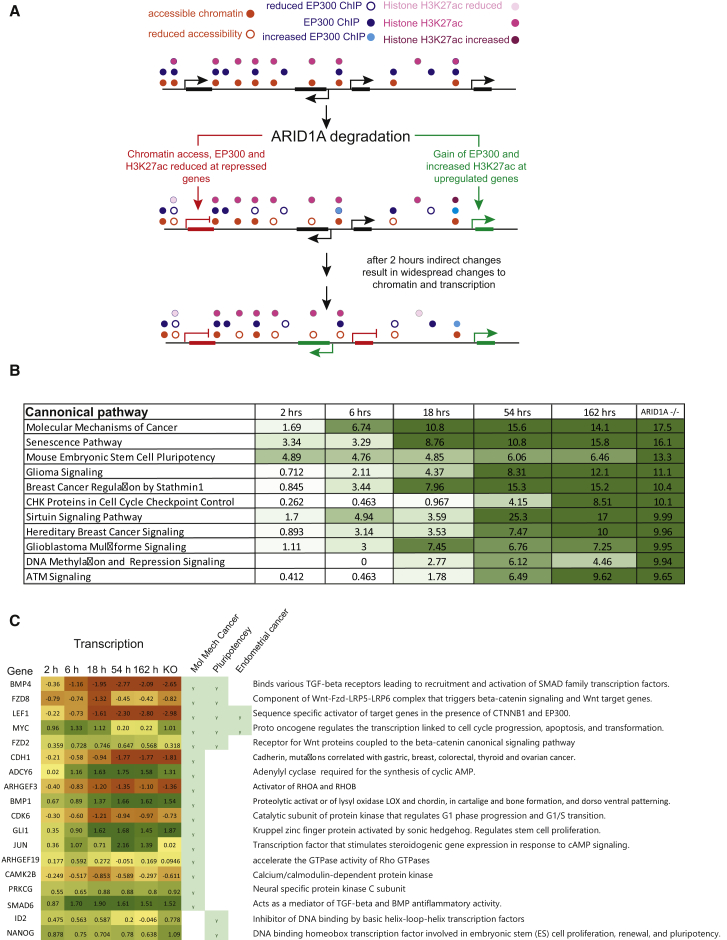
Acute and chronic changes to cancer and stem cell signaling following loss of ARID1A (A) Illustration of the two pathways by which ARID1A and EP300 act in upregulation and downregulation of gene expression. At sites adjacent to downregulated genes, chromatin accessibility (orange dots) and EP300 occupancy (blue dots) are reduced. In contrast, upregulated genes are linked to loss of EP300 occupancy in the absence of chromatin changes. These changes are enriched at genes that change expression after 2 h. Changes in expression are detected at many more genes in subsequent hours and days, but not mechanistically linked to loss of ARID1A. (B) The top pathways enriched following chronic ARID1A loss are illustrated together with the progressive engagement with these pathways over time, indicated by −log_10_ p values. (C) Genes associated with the pathways indicated that change (FDR < 0.05, fold change > log_2_ 0.5-fold) up to 6 h following loss of ARID1A are indicated together with the log_2_ transcriptional changes at all time points. The early changing components of these pathways are likely to drive downstream changes that propagate further engagement with pathways. Several early changing regulators contribute to multiple pathways. This suggests that changes to transcription of a small number of genes drives engagement with the biological pathways that shape the ARID1A^−/−^ phenotype.

Identifying genes directly linked to ARID1A-dependent changes in transcription provides the potential to unravel a network of transcriptional changes that drive adaption and ultimately the phenotypes resulting from loss of ARID1A. The genes changing after 2 h relate most strongly to pluripotency pathways (Figure 7B). This is consistent with the function of ARID1A at the binding sites of pluripotency transcription factors (Figure 2C) and reduced pluripotency following ARID1A degradation (Figure S6). Over time, the transcriptional response affects more genes (Figure 4A) and affects additional pathways. At late time points and in an ARID1A^−/−^ line, molecular mechanisms of cancer are the most strongly engaged pathway. In this respect the ARID1A degron phenocopies an ARID1A knockout line.

The strong engagement with molecular mechanisms of cancer is likely held in check by pathways such as senescence, the second most strongly enriched pathway. The correct tissue context and the presence of additional driver mutations are likely required to fully recapitulate tumor transcriptomes. As a result, the strong engagement with cancer signaling we observe is likely to be most relevant to a premalignant state. Consistent with this there is some overlap with ARID1A-driven diseases such as endometrial cancer (Le Gallo et al., 2012; Wiegand et al., 2010) (Figure 7C; Data S2). Although at later time points the differential expression of hundreds of genes contributes to engagement with these pathways, initially few genes are associated with each and several contribute to multiple pathways (Figure 7C). This raises the possibility that the phenotypes resulting from loss of ARID1A are driven by changes initiated by a handful of apex regulators and directly affected by loss of ARID1A.

## Discussion

In this study, we show how an auxin degron system (Natsume et al., 2016) is effective in targeting a specific subunit, ARID1A, of a multi-subunit complex for degradation (Figure 1). This illustrates that precision is possible using ubiquitin-mediated degradation that is likely applicable for use with different modes of targeting (Farnaby et al., 2019; Nabet et al., 2018). The use of acute depletion reveals sites at which ongoing action of SWI/SNF complexes is required to maintain chromatin accessibility. These findings are consistent with recent reports that inhibition of SMARCA4 results in loss of chromatin accessibility and transcriptional reprogramming (Iurlaro et al., 2021; Schick et al., 2021; Weber et al., 2021).

The sites where chromatin accessibility is most strongly affected are located at enhancers that include binding sites of transcription factors that play a key role in maintaining pluripotency, but the full list is diverse (Figure 2C; Data S1). In differentiated cells of different tissue types, distinct combinations of transcription factors are likely to be present at the sites where ARID1A-containing complexes act.

### BAF complexes organize clusters of nucleosomes at the binding sites for pluripotency transcription factors

Binding sites for the pluripotency transcription factors SOX2 and OCT4 are strongly enriched at rapidly lost sites of chromatin accessibility (Figure 2C). These factors not only play a key role in the establishment and maintenance of pluripotency (Takahashi and Yamanaka, 2006), but they are also observed to act as pioneer factors capable of binding to their cognate binding sites in the context of nucleosomes both *in vitro* and *in vivo* (Soufi et al., 2012; Soufi et al., 2015; Zaret and Carroll, 2011). In the absence of other factors these transcription factors both individually and in combination bind to nucleosomes at preferred locations (Dodonova et al., 2020; Li et al., 2019; Michael et al., 2020; Zhu et al., 2018). The use of precise chemical mapping reveals that histone contacts are retained at occupied binding sites for many transcription factors (Voong et al., 2016). However, the distribution of histone-DNA contacts observed in cells does not recapitulate the positional bias observed with pluripotency factors and nucleosomes in isolation. Instead, histone H4 contacts are observed to be distributed across a region spanning 70 bp either side of SOX2 and OCT4 sites (Voong et al., 2016) (Figures 3D and 3E). Our MNase-seq data highlight that nucleosome organization at the binding sites for these transcription factors is dependent on the action of ARID1A-containing complexes. The ATPase activity of SWI/SNF complexes has the ability to generate oscillating distortions to DNA (Lia et al., 2006; Zhang et al., 2006) that can direct bidirectional nucleosome motion (Harada et al., 2016). Such an activity could convert the localized nucleosome binding observed in biochemical systems lacking SWI/SNF complexes to the broader and more accessible distribution observed *in vivo*. In this case the SWI/SNF remodeled state is comprised of a nucleosome repeatedly cycling across a transcription factor-bound site. Consistent with this, chemical inhibition of SMARCA4 results in lost chromatin accessibility within a few minutes (Iurlaro et al., 2021).

The combined effects of SWI/SNF complex action and transcription factor binding result in the generation of nucleosomes that are less effectively protected from digestion by micrococcal nuclease in comparison to flanking nucleosomes (Figure 3C). These nucleosomes are highly susceptible to Tn5 transposase insertion as measured by ATAC-seq (Figure S1H). ATAC-seq is widely used to assign regulatory elements (Buenrostro et al., 2018), and the localized enrichment for the ATAC-seq signal at ARID1A-dependent factor-bound nucleosomes in comparison to that at flanking nucleosomes (Figure S1H) indicates that a key constituent of the ATAC-seq signal in mammalian cells arises from nucleosomes dynamically reconfigured by remodeling ATPases. Increased accessibility to transposase and MNase is likely to be reflected in increased access for binding of endogenous factors, including transcription factors (Agalioti et al., 2000; Côté et al., 1994; Hainer et al., 2019; Imbalzano et al., 1994; King and Klose, 2017) and HIV integrase (Kalpana et al., 1994).

The limits of distribution of histone contacts at the binding sites are proportional to the separation between adjacent nucleosomes (Figure 3E). This is anticipated if the width of the central distribution serves as a barrier (Kornberg, 1981) setting the locations of adjacent nucleosomes (Figure 3F). Consistent with this, when ARID1A is degraded, MNase-seq recovers increased reads in the vicinity of the central barrier nucleosome, and the organization of a cluster of two to six adjacent nucleosomes is reduced (Figures 3C and S1). As a result, ARID1A-containing complexes can be considered to organize a minidomain consisting of an accessible center flanked by spaced nucleosomes.

There are parallels between the function of mammalian SWI/SNF complexes at enhancers and yeast complexes at promoters. For example, the yeast RSC complex plays an important role in defining the location of the +1 nucleosome at transcriptional start sites (Ocampo et al., 2019). Furthermore, complexes containing RSC and histones are observed at wider nucleosome-depleted regions (Brahma and Henikoff, 2019). This observation links the width of a nucleosome-depleted region to the positioning of adjacent nucleosomes in a similar fashion to the wider separation between nucleosomes at ARID1A-dependent sites (Figure 3). RSC acts to set the position of a barrier at the nucleosome-depleted region (NDR), and additional nucleosome spacing enzymes act to phase nucleosomes with respect to this barrier (Prajapati et al., 2020). At the binding sites of pluripotency transcription factors, it is also possible that additional enzymes, such as SNF2H, act to set the location of flanking nucleosomes with respect to an ARID1A-dependent barrier. A difference between the two systems is that 147-bp nucleosomal fragments are recovered with 30%–50% reduced efficiency at the binding sites for mammalian pluripotency transcription factors (Figure 3), but they are absent in the nucleosome-depleted regions at yeast promoters (Chereji et al., 2017).

### Interplay between chromatin accessibility and transcription

In total, some 11,000 sites of lost chromatin are detected 2 h following degradation of ARID1A. Most of these changes occur at sites distributed across the genome and not obviously linked to differentially expressed genes (Figure 4). Relatively few changes to chromatin structure are observed to occur at promoters and when detected they tend to occur following changes to transcription (Figure 4). The lack of chromatin changes at promoters detected prior to transcriptional changes during the time course of ARID1A degradation is striking and challenges the paradigm that promoter chromatin accessibility is instructive for gene expression. This may be a specific feature of pluripotent chromatin where the potential for transcription is maintained at most genes.

### Loss of ARID1A results in genome-wide redistribution of EP300, which drives distinct pathways for transcriptional activation and repression

Consistent with previous observations, we show that the interaction of EP300 with BAF complexes requires the ARID1A subunit (Figure S3). When ARID1A is degraded, EP300 dissociates from locations where it is localized via contact with BAF complexes and is redistributed to new locations. The combined effects of ARID1A loss on chromatin accessibility and EP300 localization drive distinct pathways for transcriptional upregulation and downregulation.

EP300 dissociates from some 4,500 locations in the absence of substantial changes to chromatin. Presumably at these sites partially redundant remodeling activities such as ARID1B act to maintain chromatin accessibility (Kelso et al., 2017). These locations are not enriched adjacent to differentially expressed genes, indicating that EP300 is not required to maintain ongoing transcription, possibly due to the presence of redundant cofactors such as CBP. Sites where chromatin accessibility is lost in the absence of changes to EP300 occupancy are modestly enriched adjacent to downregulated genes. These changes are detected adjacent to 60% of downregulated genes; however, loss of chromatin accessibility is also detected adjacent to about 30% of genes that are not differentially regulated. This is best explained if ongoing transcription of many genes is robust to changes in chromatin accessibility at enhancer sites. The relatively rare cases where ongoing chromatin remodeling is required to sustain transcription are locations where EP300 is also lost rapidly. These coincident changes are detected adjacent to one third of rapidly downregulated genes. This is likely to be an underestimate, as some changes occur but are below the threshold for peak calling, and the 50-kb window for calling enhancers is crude. How the combined loss of EP300 and chromatin accessibility at enhancers affects transcription of linked genes remains to be determined. Changes to non-coding transcription appear not to be involved, as TT-seq at sites of ARID1A action shows little change over the time course (Figure S5).

In contrast to the pathway for transcriptional downregulation described above, the changes identified adjacent to upregulated genes are indirect. The release of EP300 from sites of ARID1A action may result in increased occupancy at sites where association is normally limited by the concentration of free protein and is ARID1A-independent. It is also likely that some transcription factors dissociate from sites of ARID1A action and rebind at previously unoccupied sites (Hainer et al., 2019; King and Klose, 2017). The rebinding of these factors may also contribute to the redistribution of EP300.

Across evolutionary diverse organisms inactivation of components of remodeling complexes that normally function as activators has been observed to cause upregulation and downregulation of similar numbers of genes (Chandler et al., 2015; Guo et al., 2018; Kelso et al., 2017; Martens and Winston, 2002; Mathur et al., 2017; Sudarsanam et al., 2000; Wilson et al., 2019). The redistribution of activators and co-activators provides an explanation for this and illustrates the potential for rapid but indirect effects to contribute significantly to transcriptional phenotypes.

SWI/SNF complexes have been proposed to act via redistribution of polycomb complexes (Kadoch et al., 2017; Weber et al., 2021). Enrichment for polycomb is low at sites where ARID1A regulates chromatin accessibility, and histone H3K27 methylation does not accumulate rapidly at these locations following ARID1A degradation (Figure S5). In contrast, polycomb components are enriched at a subset of the locations where EP300 occupancy increases following ARID1A degradation (Figure 5E). EP300 and other displaced activators are likely to contribute to the redistribution of polycomb components observed following loss of remodeling activity. This in turn may result in a modest and relatively slow reduction in H3K27me3 observed at sites of gained EP300 occupancy (Figure S5B).

Subunits of SWI/SNF complexes are likely to exhibit specific interactions with activators and co-activators (Sen et al., 2017). For example, inhibition of the ATPase subunits is expected to affect the action of BAF, PBAF, and GBAF forms of the SWI/SNF complex. Inactivation of ARID1A affects the subset of BAF forms of SWI/SNF complexes not containing ARID1B (Kelso et al., 2017), but it is likely to have greater effects on the distribution of EP300 than disruption of ATPase subunits. As a result, loss of different subunits will have distinct effects both on the direct activities of the complexes and the indirect redistribution of activators and co-activators. This may help to explain why different phenotypes arise from mutations to different subunits of otherwise closely related forms of chromatin remodeling complexes.

### ARID1A-dependent oncogenic pathways

EP300 association at many sites is dependent on ARID1A (Figure 5) and linked to ARID1A-dependent transcriptional changes (Figure 7A). Consistent with its related roles in oncogenesis, EP300 is mutated at high rates in several cancers that are also associated with ARID1A loss, including basal cell (23%), bladder (15%), and uterine (15%) carcinomas (Gao et al., 2013). This suggests that in some contexts loss of EP300 enhances ARID1A phenotypes or vice versa. Downstream of ARID1A and EP300 some 400 genes are differentially expressed 2 h following auxin addition, and many of these are linked to ARID1A-dependent changes to chromatin and EP300 occupancy (Figure 5). Of the first affected genes, many are associated with pathways that do not develop further over subsequent days. Nonetheless, a few act to propagate engagement with cancer pathways that become progressively enriched and ultimately dominate the phenotype of ARID1A^−/−^ knockout cells (Data S2). The identification of this hierarchy illustrates the importance of characterizing acute changes to relate genotype to phenotype. As ARID1A is a tumor suppressor, the directly affected genes represent new entry points via which it may be possible to prevent or reverse malignant phenotypes resulting from inactivation of ARID1A.

## STAR★Methods

### Key resources table

**Table:** 

REAGENT or RESOURCE	SOURCE	IDENTIFIER
**Antibodies**		

Mouse monoclonal anti-EP300	Santa Cruz Biotechnology	sc-48343; RRID:AB_62807
Mouse monoclonal anti-EP300	Abcam	ab14984; RRID:AB_301550
Rabbit polyclonal anti-H3K27ac	Abcam	ab4729; RRID:AB_2118291
Rabbit monoclonal anti-H3K27me3	Cell Signaling Technology	#9733; RRID:AB_2616029
Rabbit polyclonal anti-ARID1A	Sigma-Aldrich	HPA005456; RRID:AB_1078205
Mouse monoclonal anti-Actin, clone AC15	Sigma-Aldrich	A5441; RRID:AB_476744
Rabbit polyclonal anti-SMARCA4	Abcam	ab11064; RRID:AB_10861578

**Chemicals, peptides, and recombinant proteins**		

ESGRO® Leukemia Inhibitory Factor (LIF), 10 million units/1 mL	Sigma-Aldrich	ESG1107
auxin analog 1-naphthaleneacetic acid, NAA	Sigma-Aldrich	N0640
HAT supplement (50x)	ThermoFisher	21060017
HT supplement (100x)	ThermoFisher	11067030
Cre recombinase	Zhou et al., 2013	N/A
Flp recombinase	Zhou et al., 2013	N/A
Lipofectamine LTX Reagent with PLUS Reagent	ThermoFisher	15338030
6-thioguanine	Sigma-Aldrich	A4882
Puromycin	Sigma-Aldrich	540411
Normocin	Invivogen	ant-nr-1
4-thiouridine	Sigma-Aldrich	T4509
TRIzol	Invitrogen	15596026
MTSEA-Biotin	Biotum	90066/90066-1
Triethylammonium bicarbonate buffer pH 8.5, TEAB	Sigma-Aldrich	T7408
Pierce TCEP, Tris(2-carboxyethyl)phosphine hydrochloride	ThermoFisher	20490
Pierce Universal nuclease	Pierce	88702
Iodoacetamide	Sigma-Aldrich	I6125
Lysyl endopeptidase, Lys-C	FUJIFILM Wako Pure Chemical Corporation	125-05061
Pierce Trypsin	ThermoFisher	90058
Sera-Mag Speed Beads	VWR	CAT# 09-981-121
Sera-Mag Speed Beads	VWR	CAT# 09-981-123
C7BzO	Sigma-Aldrich	C0856
Roche, Nuclease S7, Micrococcal nuclease	Roche	50-100-3364

**Critical commercial assays**		

Leukocyte Alkaline Phosphatase Kit	Sigma-Aldrich	86R
TURBO DNA-free kit	ThermoFisher	AM1907
μMACS Streptavidin MicroBeads	Miltenyi Biotec	130-074-101
NEBNext Ultra II Directional RNA library prep Kit for Illumina with Sample Purification	New England Biolabs	E7765S
NEBNext Multiplex Oligos for Illumina (Index Primers Set)	New England Biolabs	E7710S
NEBNext Ultra II DNA Library Prep with Sample Purification Beads	New England Biolabs	E7103L
NEBNext Multiplex Oligos for Illumina (Dual Index Primers Set)	New England Biolabs	E7600S
Nextera Index kit	Illumina	15055289
Nextera DNA library Prep Kit	Illumina	15028121
NEBNext High Fidelity 2X PCR Master Mix	New England Biolabs	M0541L
EZQ Protein Quantification Kit	ThermoFisher	R33200
C18 Sep-Pak cartridges	Waters	WAT036945
CBQCA Protein Quantitation Kit	ThermoFisher	C6667
TMT10plex Isobaric Label Reagent Set	ThermoFisher	90111
Dynabeads® Protein A for Immunoprecipitation	ThermoFisher	10002D
Dynabeads® Protein G for Immunoprecipitation	ThermoFisher	10004D
Agencourt AMPure XP beads	beckman coulter	A63881
PreCR Repair Mix	New England Biolabs	M0309
SimpleChIP Enzymatic Chromatin IP Kit	Cell Signaling Technologies	9003S
NEBNext End Prep	New England Biolabs	E7442
NEBNext Adaptor for Illumina	New England Biolabs	E7335
NEBNext Q5 Hot Start HiFi Master Mix	New England Biolabs	M0543S

**Deposited data**		

Raw NGS data	This paper	GEO: GSE183278; BioProject: PRJNA662266
Processed NGS data (bigwig files)	This paper	GEO: GSE183278
Proteomics data ARID1A-IP-MS	This paper	PRIDE Project accession: PXD021824
Proteomics data EP300-IP-MS	This paper	PRIDE Project accession: PXD021624
Proteomics data SMARCA4-IP-MS	This paper	PRIDE Project accession: PXD021631
Proteomics data TMT analysis	This paper	PRIDE Project accession: PXD021636
ChIP data for CTCF	Handoko et al., 2011	GSE28247, SRR172853-SRR172854
ChIP data for REST	Whyte et al., 2012	GSE27841, SRR122473
ChIP data for SOX2, OCT4, NANOG	Whyte et al., 2013	GSE44286, SRR713341, SRR713340, SRR713342
ChIP data for KLF4	Chen et al., 2008	GSE11431, SRR002000-SRR002003, SRR002016-SRR002019, SRR001988-SRR001991
ChIP data for TAF1	Liu et al., 2011	GSE30959
ChIP data for MAFK	Snyder lab, 2014	Mouse Encode ENCFF599GJE
ChIP data for RNAPolIIS5P	Brookes et al., 2012	GSE34520, SRR391032-SRR391033
ChIP data for H3K4me1, H3K4me3	Cruz-Molina et al., 2017	GSE89211, SRR4453260 SRR4453261
ChIP data for BRD4	Young Lab, Whitehead Institute for Biomedical Research	GSE36561, SRR500928
ChIP data for MED1	Kagey et al., 2010	GSE22562, SRR058987, SRR058988
ChIP data for MLL3/MLL4	Dorighi et al., 2017	GSE98063, SRR5466740-SRR5466741
ChIP data for SMARCA4	de Dieuleveult et al., 2016	GSE64825, SRR1747925, SRR1747926
ChIP data for CBP	Hnisz et al., 2013	SRR1014797
Chip Data for EZH2	Mu et al., 2018	GSE123174, SRR8267520-SRR8267521
ChIP data for H3 K27me3	Cruz-Molina et al., 2017	GSE89211, SRR4453259

**Experimental models: Cell lines**		

E14TG2a.4 mESC, *Mus musculus* strain 129/Ola	Zhou et al., 2013	N/A
ET905, mES E14TG2a.4 TIR1 clone #905	This paper	N/A
AAG57, mES E14TG2a.4 TIR1, ARID1A-mini-auxin-inducible-GFP-tagged clone #57	This paper	N/A
ARID1A-ko, mES E14TG2a.4 TIR1, homozygous ARID1A ko #53	This paper	N/A

**Oligonucleotides**		

gRNAs site for the C-terminal ARID1A tagging sense: CTGATGAACTCATTGGTTTC	This paper	N/A
gRNAs site for the C-terminal ARID1A tagging antisense: CGGCTGTCATGACTGGCCAA	This paper	N/A
gRNA site of mouse ARID1A ko, sense: GTGTGGAGTCTGGGACCCATA	This paper	N/A
gRNA site of mouse ARID1A ko, antisense: GCGGTACCCCATGACCATGCA	This paper	N/A

**Recombinant DNA**		

pAW5-EF1α-OsTIR1	This study	N/A
pBS-mARID1A-AID-GFP-donor	This study	N/A
pX335-gRNA-C-terminal-ARID1A	This study	N/A
pU6 puro-gRNA-C-terminal-ARID1A	This study	N/A
pBabeD-U6-mARID1A-gRNA-KO-ex2 s	This study	N/A
pX335-mARID1A-gRNA-KO-ex2-as	This study	N/A

**Software and algorithms**		

FastQC		https://www.bioinformatics.babraham.ac.uk/projects/fastqc/ RRID:SCR_014583
Trim Galore		https://www.bioinformatics.babraham.ac.uk/projects/trim_galore/ RRID:SCR_011847
cutadapt	Martin, 2011	https://cutadapt.readthedocs.io/en/stable/ RRID:SCR_011841
Trimmomatic	Bolger et al., 2014	N/A
MaxQuant	Cox and Mann, 2008	https://maxquant.net/maxquant/ RRID:SCR_014485
Perseus Software	Tyanova et al., 2016	RRID:SCR_015753
Bowtie2	Langmead and Salzberg, 2012	http://bowtie-bio.sourceforge.net/bowtie2/index.shtml RRID:SCR_005476
Samtools	Li et al., 2009	https://htslib.org/ RRID:SCR_002105
MACS2	Zhang et al., 2008	https://github.com/macs3-project/MACS RRID:SCR_013291
STAR aligner	Dobin et al., 2013	https://github.com/alexdobin/STAR RRID:SCR_015899
deepTools	Ramírez et al., 2016	https://github.com/deeptools/deepTools RRID:SCR_016366
BEDTools	Quinlan and Hall, 2010	https://github.com/arq5x/bedtools2 RRID:SCR_006646
edgeR	Robinson et al., 2010; McCarthy et al., 2012	http://bioconductor.org/packages/release/bioc/html/edgeR.html RRID:SCR_012802
Diffbind	Ross-Innes et al., 2012	http://bioconductor.org/packages/release/bioc/html/DiffBind.html RRID:SCR_012918

### Resource availability

#### Lead contact

Further information and request for resources should be directed to and will be fulfilled by the lead contact, Tom Owen-Hughes (t.a.owenhughes@dundee.ac.uk).

#### Materials availability

Plasmids and cell lines generated in this study can be requested without restriction upon an agreement with our institute’s material transfer agreement (MTA).

### Experimental model and subject details

#### Cell culture and ARID1A depletion

All cell lines used in this study originate from E14TG2a.4 mESC (mouse Embryonic Stem Cells, *Mus musculus* strain 129/Ola, male) with integrated gene targeting vector pAW2 at the ROSA26 locus to enable recombinase-mediated cassette exchange (Zhou et al., 2013). mESC cells were grown in G-MEM medium (ThermoFisher) supplemented with 5% embryonic stem cell-qualified FBS (ThermoFisher), 5% Knockout Serum Replacement (ThermoFisher), 1x non-essential amino acids (ThermoFisher), 1x sodium pyruvate (ThermoFisher), 100 μM β-mercaptoethanol (Sigma-Aldrich) and 1000 U/mL LIF (Millipore). Cells were sub-cultured every two days into dishes coated with 0.1% gelatine and kept at 37°C in a 5% CO_2_ water-saturated incubator.

For depletion of ARID1A, cells were seeded at ∼20,000 cells per cm^2^ and 500 μM auxin analog 1-naphthaleneacetic acid (NAA, Sigma-Aldrich) in 1M sodium hydroxide was added for the indicated time before harvesting the cells after two days of growing.

#### Integration of TIR1 at the ROSA26 locus

2x10^7^ E14TG2a.4 AW cells (Zhou et al., 2013) were transfected with a plasmid encoding Cre-recombinase (10 μg) and 30 μg pAW5-EF1α-OsTIR1 30 μg by electroporation with the Neon transfection system (ThermoFisher) at 1200 V, 30 ms, 1 pulse. 24-48 hours post-electroporation, the medium was changed and supplemented with HAT (ThermoFisher). Cells were grown under HAT selection until colonies could be picked. Colonies were expanded to 6-well plates containing medium supplemented with HT (ThermoFisher), and isolated clones were tested for neomycin-sensitivity. Correctly recombined clones (3.6x10^5^ cells in a 6-well) were reverse transfected with Flp recombinase (1.2 μg) using Lipofectamine LTX (3 μL) with Plus reagent (1.2 μL) (ThermoFisher), and the medium was replaced after 4-6 hours to minimize toxicity. Cells were grown to confluency, harvested and seeded in duplicate at 5000 cells per 10 cm dish. After 48 hours, medium was supplemented with 10 μM 6-thioguanine (Sigma-Aldrich) and left until colonies formed. As before, colonies were isolated and expanded containing normal medium. Correct insertion of TIR1 was verified by western blotting.

#### Tagging ARID1A and establishing knockout by CRISPR in mouse ESCs

For tagging ARID1A cell lines expressing EF1α-driven TIR1 were reverse transfected in the following manner: 3x10^5^ cells were seeded into a 6-well dish. 480 ng donor template (800 bp homology arms surrounding the mAID-GFP insert) and 160 ng each of gRNA plasmids were mixed in 200 μL serum-free medium, and 4 μL polyethyleneimine (1 mg/mL, Polysciences Inc.) was added. The solution was vortexed, incubated at room temperature for 30 min, and then added drop-wise to the cells. The following day, puromycin (Sigma-Aldrich) was added at 0.5 μg/mL. After 24 hours the medium was replaced and fresh puromycin added at 1 μg/mL. The following day, the medium was replaced, and cells allowed to recover. GFP-positive cells were sorted using FACS as a pool of 6000-8000 cells on the BD Influx. Cells were seeded to 6 cm dishes (containing medium supplemented with 1x penicillin-streptomycin (ThermoFisher), 1x L-glutamine (ThermoFisher) and optionally, 0.1 mg/mL normocin (Invivogen) at approximately 1500 cells per dish and left until colonies formed. Individual colonies were picked and expanded. Clones were tested for construct integration using PCR and verified for tag insertion by western blotting. Antibodies used in this study for western blots were anti-ARID1A (Millipore, HPA005456) and anti-Actin (Sigma, clone AC15, A5441).

To generate a knockout ARID1A cells line, mESC expressing EF1α-driven TIR1 were reverse transfected and selected with puromycin as described above but using 400 ng pBabeD-U6-mARID1A-gRNA-KO-ex2 s) and 400 ng pX335-mARID1A-gRNA-KO-ex2-as. After antibiotic selection, cells were seeded at low density (5000 cells per 10 cm dish) and grown until colonies had formed. Colonies were picked and expanded for screening by western blotting. To confirm the knockout of ARID1A, the genomic DNA of exon2 was cloned and sequenced in bacteria using the StrataClone Blunt PCR cloning kit (Agilent). All clones sequenced (6) showed the same 13 bp deletion causing an early stop codon after amino acid 430 (isoformX1).

### Method details

#### Plasmid construction

The plasmid (pAW5-EF1α-OsTIR1) encoding the EF1α promoter controlling the OsTIR1 gene in the pAW5 vector (Watson et al., 2008) was cloned using SLIC cloning.

To generate the donor plasmid for CRISPR-tagging ARID1A C-terminal (pBS-mARID1A-AID-GFP-donor), 800 bp homology arms with additional restriction site were ordered from Biomatik (pBS-mARID1A-donor). GFP with an additional N-terminal linker sequence (coding for SEFGGGSGGGSG) was introduced to pBS-mARID1A-donor using EcoRI and PstI restriction sites. The mini-AID fragment was amplified by PCR and cloned in the EcoRI site resulting in the final donor plasmid. gRNAs for the C-terminal ARID1A tagging (sense: CTGATGAACTCATTGGTTTC, antisense: CGGCTGTCATGACTGGCCAA) were cloned into pX335 (also coding for the CAS9 nickase: SpCas9D10A) and pU6 puro (MRC, Dundee, DU46129), respectively.

To knockout ARID1A in mESC, gRNAs targeting exon2 were cloned by the MRC Dundee cloning service: pBabeD-U6-mARID1A-gRNA-KO-ex2 s (GTGTGGAGTCTGGGACCCATA) and pX335-mARID1A-gRNA-KO-ex2-as (GCGGTACCCCATGACCATGCA).

#### Alkaline phosphatase staining

5x10^3^ degron tagged mESC and ARID1A knock out mESC were grown for six days to form colonies in the absence or presence of LIF and 0.5 μM Auxin-analog NAA in 6 well dishes. Cells were fixed using the Leukocyte Alkaline Phosphatase Kit (Sigma-Aldrich, 86R) for one minute with 3% formaldehyde/6.75 mM Citrate Solution/65% acetone and washed twice with water. Colonies were stained for 15 minutes using sodium nitrite/FRV-Alkaline and Naphthol AS-BI according to the manufacturer.

#### Transient transciptome sequencing (TT-seq) of ARID1A-depleted mESCs

80 to 90% confluent cells in 10 cm dishes were labeled in media for 10 min with 500 μM 4-thiouridine (4sU, Sigma-Aldrich) at 37°C. One 10 cm dish per condition and replicate was used. Media was discarded and RNA was extracted using TRIzol (Invitrogen) following the manufacturers’ instructions. RNA was treated with DNase using the TURBO DNA-free kit (Ambion) according to the manufacturer but increasing the amount of DNase to 6U and the inactivation reagent to 0.2 volumes. After DNase treatment the beads were removed using QIAshredder columns (QIAGEN). 75 μg RNA in 100 μl water was fragmented employing a Biorupter pico (E4) 20 × 30 s on/off on high power and checked on the TapeStation Bioanalyser (Agilent). Fragmented RNA was labeled with MTSEA-Biotin (final 300 nM, Biotium) in 1x TE buffer (pH7.4) for 1.5hrs at room temperature. Unincorporated biotin was removed by extracting the RNA twice with chloroform and precipitating the RNA. After resuspending, biotinylated RNA was isolated using 100 μl μMACS Streptavidin MicroBeads (Miltenyi Biotec), washed with 2.7 mL Wash Buffer (100 mM Tris HCl pH 7.5, 10 mM EDTA, 1 M NaCl, 0.1% Tween20) at 65°C and followed by a wash with 2.7 mL Wash Buffer at room temperature. Elution was performed using twice 100 μl 100 mM DTT. Nascent RNA was recovered using the RNeasy MinElute Cleanup Kit (QIAGEN) and eluted in 20 μl water. Libraries were prepared using the NEB Next Ultra II Directional RNA library prep Kit for Illumina according to the protocol. Each replicate was sequenced with ∼30 Mio reads per sample with four replicates at each time point on the Illumina Nextseq 500 (TCGA Dundee) and NovaSeq 6000 (Novogene, HK).

#### ATAC-seq of ARID1A-depleted cell lines

ATAC-seq libraries were generated following the Omni-ATAC protocol (Corces, M.R. et al. *Protocol Exchange* https://dx.doi.org/10.1038/protex.2017.096 (2017)) without enrichment for viable cells. Depending on the cell line used, cell viability was between 82 and 95%. In short, cells were trypsinised and pelleted. The cell pellet was resuspended in ice cold PBS and counted. 50,000 cells were added to 1 mL RSB buffer (10 mM Tris HCl pH 7.5, 10 mm NaCl, 3 mM MgCl_2_) and spun down for 5 min 500 x g at 4°C. The cell pellet was resuspended in 50 μl RSB plus 0.1% Tween20, 0.1% NP-40, 0.01% digitonin and incubated on ice for 3 minutes. The cells were spun down and resuspended in 1 mL RSB with 0.1% Tween20 before spun again. The resulting pellet was carefully resuspended in 16.5 μl PBS, 0.5 μl 1% digitonin, 0.5 μl 10% Tween20, 5 μl water, 25 μl Illumina buffer and 2.5 μl transposase enzyme and incubated for 30 minutes at 37°C while mixing with 1000rpm. After the transposase reaction, the DNA was purified using MinElute PCR purification spin columns (QIAGEN) and eluted in 10 μL EB. For library amplification, 10 μl fragmented DNA was amplified in a reaction with 2.5 μl N70x index (Nextera Index, Illumina), 2.5 μl N50x index (Nextera Index, Illumina), 25 μl NEBNext High Fidelity PCR Master Mix and 10 μl water (5min 72°C, 30sec 98°C, 10 cycles 10sec 98°C, 30sec 63°C, 1min 72°C). Amplified barcoded DNA was purified over MinElute PCR purification spin columns (QIAGEN) and eluted in 10 μL EB. The DNA concentration was measured with the Qubit High Sensitivity assay (ThermoFisher) and the fragment sizes were determined on the TapeStation Bioanalyser (Agilent). Samples were pooled in equimolar ratios to obtain at least 22 nM total DNA. The pooled library was subjected to a dual size selection using Agencourt AMPure XP beads using 0.45x and 1.4x beads:sample to enrich for fragments between 180 bp and 800 bp. Multiplexed libraries were sequenced with 2x150 bp paired-end reads by Novogene (HK) with ∼30 Mio reads for each of three replicates at each time point.

#### Peak calling and data analysis of TT-seq data

Fastq files were trimmed using Trim Galore 0.5.0 and CutAdapt 2.4 with default parameters. Sequence reads were aligned using STAR to the reference indexed GRCm38 genome including splice junctions (parameters: -outSAMtype BAM SortedByCoordinate -outSAMattributes All –quantMode GeneCounts TranscriptomeSAM) –outSAMFlagOR 243 –outSAMFlagAND 3852). Differential expression was calculated using the edgeR package in R studio. After filtering the reads for at least 5 counts per gene in three replicates, reads were normalized using the inbuilt TMM normalization and batch correction was performed. F-test was used for the pairwise comparison of reads at certain time points to time point 0 hours (no ARID1A depletion). Genome coordinates and gene names for differential genes were determined querying Ensembl Biomart. Genes were selected as differentially expressed at time point based on an FDR of < 0.05. The subset of not changing genes used in Figures 4 and 6 is selected based on no change with FDR < 0.05 then ranking the top 500 with the lowest maximum fold-change squared at any time point. When calling changes to ATAC peaks and ChIP peaks adjacent to differentially expressed genes, changes were called within a 50 kb region flanking each gene but excluding 500 bp either side of the TSS. Histone genes were excluded from this analysis as many genes reside within 50 kb.

#### Peak calling and data analysis for ATAC-seq

Fastq files were trimmed using trimmomatic-0.36 (CROP: 66) and aligned to the mouse mm10 genome using bowtie2 with the parameter –X 1000 –x mm10. Peaks were called using MACS2 *callpeak* function with the following parameters: -t “$1.”bam -f BAM -n “$1”_MACS -g 1.87e9 -q 0.05–broad -B. Differential peaks were obtained using DiffBind, doing pairwise comparison of two time points. When performing *dba.count*, a minOverlap was set to 3, requiring a peak to be observed in at least 3 datasets in order to be retained. Differential peaks were called using the edgeR method during *dba.analyze*. Of the differentially called peaks, a second filtering step was performed to retain only peaks that met an FDR < 0.00001 and a scores.fold > 0.58 (equal to a fold change > 1.5). Non-changing peaks were obtained from the DiffBind consensus peak set, with all differential peaks removed (all time points, no extra thresholding). The *bedtools intersect* function was used to call overlap of ATAC-seq peaks with ChIP-seq data (accessed from the GEO database) and chromatin states (obtained from https://github.com/guifengwei/ChromHMM_mESC_mm10).

MEME-ChIP (Motif Analysis of Large Nucelotide Datasets) (Machanick and Bailey, 2011) was performed using randomly subsampled non-differential peaks to the same sample-size as differential peaks as control peaks and the following settings: -dna -order 1 -ccut 500 -meme-mod zoops -meme-minw 6 -meme-maxw 30 -meme-nmotifs 10 -meme-p 10 -meme-searchsize 10000000 -dreme-e 0.05 -dreme-m 50 -centrimo-score 5.0 -centrimo-ethresh 10.0 -spamo-skip –db HOCOMOCOv11_full_MOUSE_mono_meme_format.mem.

#### ChIP-seq in ARID1A degron cells

ARID1A-tagged degron mESC with auxin treatment according to the time point indicated were crosslinked with 1% formaldehyde for 10 min and quenched for 5 min with 125 mM glycine at room temperature. After washing cells twice with ice-cold PBS, cell pellets were flash frozen in liquid nitrogen and stored at −80°C.

For histone ChIP, 3.2 x10^7^ cells were used per time point and replicate. Cells were lysed in lysis buffer containing 1% SDS, 10 mM EDTA, 50 mM Tris pH 8.1 and protease inhibitors. Cells were sonicated for 60 cycles (7.5 min total sonication time) at low setting using a Bioruptor (Diagenode). Sonicated lysates were then cleared by centrifugation for 10 min at high speed, diluted 1/10 with dilution buffer (1% Triton X-100, 2 mM EDTA, 150 mM NaCl, 20 mM Tris at pH 8.1, 0.1% Brij-35) and incubated with 2 μg of anti H3K27ac antibody (Abcam, ab4729) or 10 μL anti H3K27me3 (CST, #9733) overnight at 4°C, respectively. 50 μL of Protein A Dynabeads (Life Technologies) were pre-incubated with 0.5% (w/v) BSA in PBS overnight. To capture antibody-bound protein-DNA complexes, lysates were incubated with the prepared beads for 4 hr and subsequently washed twice with wash buffer I (0.1% SDS, 1% Triton X-100, 2 mM EDTA pH 8.0, 20 mM Tris pH 8.1, 150 mM NaCl), wash buffer II (0.1% SDS, 1% Triton X-100, 2 mM EDTA pH 8.0, 20 mM Tris pH 8.1, 500 mM NaCl), wash buffer III (0.25 mM LiCl, 1% NP-40, 1% sodium deoxycholate, 1 mM EDTA pH 8.0, 10 mM Tris pH 8.1) and TE buffer (10 mM Tris pH 8.1, 1 mM EDTA pH 8.0) in the cold. DNA was eluted using 1% SDS, 100 mM NaHCO_3_ and reverse-crosslinked overnight before the clean up using AMpure XP beads.

For EP300 ChIP, 3.8 × 10^7^ cells were used per time point and replicate. Cells were lysed in MNase lysis buffer (10 mM Tris pH 7.5, 10 mM NaCl, 3mM MgCl_2_, 0.5% NP40, 0.15 mM spermine, 0.5 mM spermidine, MiniComplete plus, Roche) and digested in MNase digest buffer (10 mM Tris pH 7.5, 60 mM NaCl, 1 mM CaCl_2_, 0.15 mM spermine, 0.5 mM spermidine, 0.5 mM DTT) using 36 units of S7 MNase (Roche) for 20 minutes at 37°C. The digest was stopped by adding 50 mM EDTA and cells were spun down. Cell pellets were resuspended in ChIP buffer (SimpleChIP Enzymatic Chromatin IP Kit, CST). ChIP was performed using 5 μg anti-EP300 antibody (sc-48343, Santa Cruz) and the manufacturer’s protocol was followed for the immunoprecipitation, DNA reverse crosslinking and clean up.

Libraries were prepared using the NEBNext Ultra II DNA Library Prep Kit for Illumina with multiplex oligos (#E7600). Libraries were pooled equimolar and sequenced with 2x150 bp paired-end reads by Novogene (UK) with ∼20 Mio reads for each of four replicates (two biological and two technical) were processed for each condition except EP300 at zero hours which is based on two replicates.

#### Peak calling and data analysis for ChIP-seq

Fastq files were trimmed using Trim Galore version: 0.5.0 with paired-end trimming mode, default settings (Cutadapt version: 2.4) and aligned to the mouse mm10 genome using bowtie2 with the parameter –X 1000 –x mm10. Peaks were called using MACS2 *callpeak* function with the following parameters: -c input.bam -t “$1.”bam -f BAMPE -n “$1” -g 1.87e9 -q 0.05 (H3K27ac and H3K27me3) or -q 0.2 (EP300), with the option of broad peaks for H3K27me3. Differential peaks were obtained using DiffBind, doing pairwise comparison of two time points. When performing *dba.count*, a minOverlap was set to 2, requiring a peak to be observed in at least 2 datasets in order to be retained. Differential peaks were called using the edgeR method during *dba.analyze*. Of the differentially called peaks, a second filtering step was performed to retain only peaks that met an FDR < 0.00001 and a scores.fold > 0.58 (equal to a fold change > 1.5). The key resource table provides links to datasets used in comparisons with data generated in this study.

#### Nuc-seq after ARID1A depletion

ARID1A depletion was induced in ARID1A-tagged degron mESC with 0.5 μM auxin for the indicated time points. Cells were crosslinked with 1% formaldehyde for 10 min and quenched for 5 min with 125 mM glycine at room temperature. After washing cells with cold PBS, cells were lysed using cold NP40-lysis buffer (10 mM Tris pH 7.5, 10 mM NaCl, 3 mM MgCl_2_, 0.5% NP40, 0.15 mM spermine, 0.5 mM spermidine) for 5 min on ice. Cells were pelleted and washed with MNase digestion buffer (10 mM Tris pH 7.5, 15 mM NaCl, 60 mM KCl, 1 mM CaCl_2_, 0.15 mM spermine, 0.5 mM spermidine) and resuspended in 50 μL MNase digestion buffer. For the digest, 7.5 units MNase S7 (Roche) were added to ∼4 × 10^6^ cells and incubated for 14 min at 37°C. The digest was stopped adding 1/10 vol 10% SDS and 1/10 vol 250 mM EDTA. 0.24 M NaCl and 10 μg RNase A was added to reverse the crosslinking at 65°C for 2 hr. The samples were treated with 20 μg proteinase K overnight at 65°C, followed by phenol-chloroform extraction and purification using NEB Monarch PCR purification kit. The resulting DNA fragments were used for Illumina library preparations using PreCR Repair Mix (NEB M0309), followed by NEBNext End Prep (NEB E7442) and NEBNext Adaptor for Illumina (NEB E7335) ligation according to the manufacturer’s protocol. DNA was purified using AMpure beads and amplified by PCR using NEBNext Q5 Hot Start HiFi Master Mix (NEB E6625AA), NEB Universal Primer and NEB Index Primer for 7 cycles. PCR was cleaned up using AMpure beads and sequenced on Illumina NextSeq 500 with 2x 50 nt paired-end reads. Two replicates of each condition were sequenced to a depth of 220 million reads.

#### Nuc-seq analysis

Fastq files were aligned to the mouse mm10 genome using bowtie2 with the parameter –X 1000 –x mm10. Files were concatenated from three different runs with two replicates each. The midpoints of uniquely mapped nucleosomal reads were used for further analysis. A 1 kb region flanking either side of transcription factor (TF) motifs that are bound by the relevant TF based on overlap with ChIP data. The centers of nucleosomal fragments were summed for each base pair across the 2 kb window at each TF binding motif. The enrichment value at each base was calculated by dividing the read count by the number of TF binding sites and the total number of reads in the experiment. For comparison to chemical nucleosome mapping a similar calculation was made using the nucleosome center positioning score dataset GEO:GSE82127 (Voong et al., 2016). Prior to plotting data was smoothed using a 30 bp moving average.

#### Immunoprecipitation-mass spectrometry

mESC pellets fresh or snap frozen were used for immunoprecipitation. 2 μg antibody (EP300 – Abcam 14984, ARID1A - HPA005456 Sigma-Aldrich, SMARCA4 – Abcam 110641) was DMP-crosslinked to Dynabeads Protein A or G (Invitrogen). Cells were lysed on ice in buffer containing 50 mM Tris HCl pH 7.5, 150 mM NaCl, 0.5% C7BzO and protease inhibitors. 750 μg of protein lysate was incubated with crosslinked beads for 4 h at 4 °C. Immunoprecipitations were washed with 50 mM Tris HCl pH 7.5, 750 mM NaCl and eluted in 7.5% SDS, 10 mM TCEP, 50 mM triethylammonium bicarbonate buffer pH 8.5 (TEAB). Alkylation was performed for 15 min with 20 mM iodoacetamide at 25°C in the dark and proteins were cleaned up using the SP3 (VWR, Sera-Mag Speed Beads 1:1, CAT# 09-981-121, 09-981-123, rinsed with water). SP3 beads mix (20 μg/μl) was added to the immunoprecipitation (1 μg/μl). Sample was acidified to pH 2-3 using 10% formic acid and bound to beads with one volume 100% acetonitrile. Beads were rinsed twice with 70% ethanol followed once by 100% acetonitrile. Elution was performed in 50 mM TEAB and digestion with 1 μg trypsin (Pierce) per sample overnight at 37 °C. SP3 beads were not removed for peptide clean up. Resulting peptides were bound to the SP3 beads by addition of one volume acetonitrile and washed twice with 100% acetonitrile. After drying, elution was performed using 2% DMSO in water and peptides were vacuum dried. Data dependent acquisition was carried out on the Orbitrap Velos in collision-induced dissociation (CID) mode. The data were processed using MaxQuant (v.1.5.0.25). The t test differences between means for protein abundance of cells with and without ARID1A depletion were calculated using LFQ intensities and plotted versus log_10_ *P value*s determined by two-tailed t test (Perseus Software, v.1.6.1.3). For Figure S3 the t test differences were also determined between means for protein abundance in IPs using a specific antibody or the IgG control. Each experiment was performed in quadruplicates.

#### TMT-labeling proteomics

Total proteome profiling of mESC before (0 hours) and after 2 hours depletion of ARID1A was performed by 10-plex Tandem Mass Tag (TMT)-labeling. Cell pellets were washed with ice cold PBS and re-dissolved in 200 μL of urea buffer (8 M urea in 100 mM TEAB) and mixed at room temperature for 15 minutes. Cellular DNA was sheared using universal nuclease (Pierce) at 37°C for 15 minutes. The proteins were reduced using 25 mM TCEP for 30 minutes at room temperature, then alkylated in the dark for 30 minutes using 50 mM iodoacetamide. Total protein was quantified using the EZQ assay (Invitrogen). Lysates were digested with lysyl endopeptidase, Lys-C (Wako), enzyme to substrate ratio of 1:50 (w/w), in 100 mM TEAB overnight at 37°C, followed by digestion with trypsin (Pierce) at the same concentration overnight at 37°C. Digestion was stopped by acidification with 1% TFA (final). Peptides were desalted using C18 Sep-Pak cartridges (Waters) following manufacturer’s instructions.

The dried peptides were re-dissolved in 50 μL 100 mM TEAB and the concentration was measured using a fluorescent assay (CBQCA Protein Quantitation Kit, Invitrogen). 100 μg of peptides were labeled with a different TMT tag (0.8 mg TMT10plex label reagent (ThermoFisher) dissolved in 41 μL of dry acetonitrile), for 2 h at room temperature. After incubation, the labeling reaction was quenched using 8 μL 5% hydroxylamine for 30 min. The samples were mixed at equimolar ratios and vacuum dried.

The TMT samples were fractionated and analyzed by MRC Unit, School of Life Sciences, University of Dundee. Fractionation was performed by pH reverse-phase (RP) chromatography. Peptides were separated into 96 fractions that were concatenated into 30 fractions and run on a Lumos Mass Spectrometer. The data was processed and analyzed using MaxQuant (v. 1.6.6.0) and Perseus (v. 1 0.6.3.2). The total proteome profiling for 0 h depletion of ARID1A in mESC was performed in quadruplicates, the 2 h depletion in triplicates.

### Quantification and statistical analysis

False discovery rate (FDR) is calculated by the ratio of the number of false positive values to the number of total positive values.

The probability distribution function was calculated as

$$\frac{\left(\begin{array}{l} M\\ x \end{array}\right)\left(\begin{array}{l} N-M\\ n-x \end{array}\right)}{\left(\begin{array}{l} N\\ n \end{array}\right)}$$

Where x = changes occurring at differentially regulated gene cohort, n = number of genes in differentially regulated cohort (This one fifth of the genes changing at each time point; 389 genes after 2 hours, 1169 genes after 6 hours, 908 genes after 18 hours, 3089 genes after 54 hours and 989 genes after 162 hours), M = the total number of changes detected in all genes called as transcribed from TT-seq data, N the total number of genes called as transcribed from TT-seq data (12741 genes). The numbers of changes occurring in each cohort is plotted as a separate panel related to each probability distribution.

## Data Availability

The data generated by Next generation sequencing: Transient Transcriptome (TT-seq), Assay for Transposase-Accessible Chromatin (ATAC-seq), Chromatin Immunopreciptiation (ChIP-seq), Micrococcal Nuclease (Nuc-seq) sequencing in this study have been deposited at NCBI and are publicly available as of the date of publication, GEO: GSE183278. Mass spectrometry data have been deposited in the ProteomeXchange Consortium via the PRIDE partner repository with the dataset identifiers PRIDE Project: PXD021824, PXD021624, PXD021631, PXD021636. This paper does not report original code. Any additional information required to reanalyze the data reported in this paper is available from the lead contact upon request.

## Supplemental information

Document S1. Figures S1–S6

Data S1. E values measuring enrichment of transcription factor motifs, related to Figure 2

Data S2. Progressive engagement with cancer signaling following degradation of ARID1A, related to Figure 7 Document S2. Article plus supplemental information

Document S2. Article plus supplemental information
