# Supplementary material for: Acute depletion of the ARID1A subunit of SWI/SNF complexes reveals distinct pathways for activation and repression of transcription
Source: Cell Rep. 2021 Nov 2;37(5):109943. doi: 10.1016/j.celrep.2021.109943 (PMC8578704; doi:10.1016/j.celrep.2021.109943)
Supplement: Document S1. Figures S1–S6 [file mmc1.pdf]

Cell Reports, Volume *37*

Supplemental information

Acute depletion of the ARID1A subunit of SWI/SNF complexes reveals distinct pathways for activation and repression of transcription

Seraina Blümli, Nicola Wiechens, Meng-Ying Wu, Vijender Singh, Marek Gierlinski, Gabriele Schweikert, Nick Gilbert, Catherine Naughton, Ramasubramanian Sundaramoorthy, Joby Varghese, Robert Gourlay, Renata Soares, David Clark, and Tom Owen-Hughes

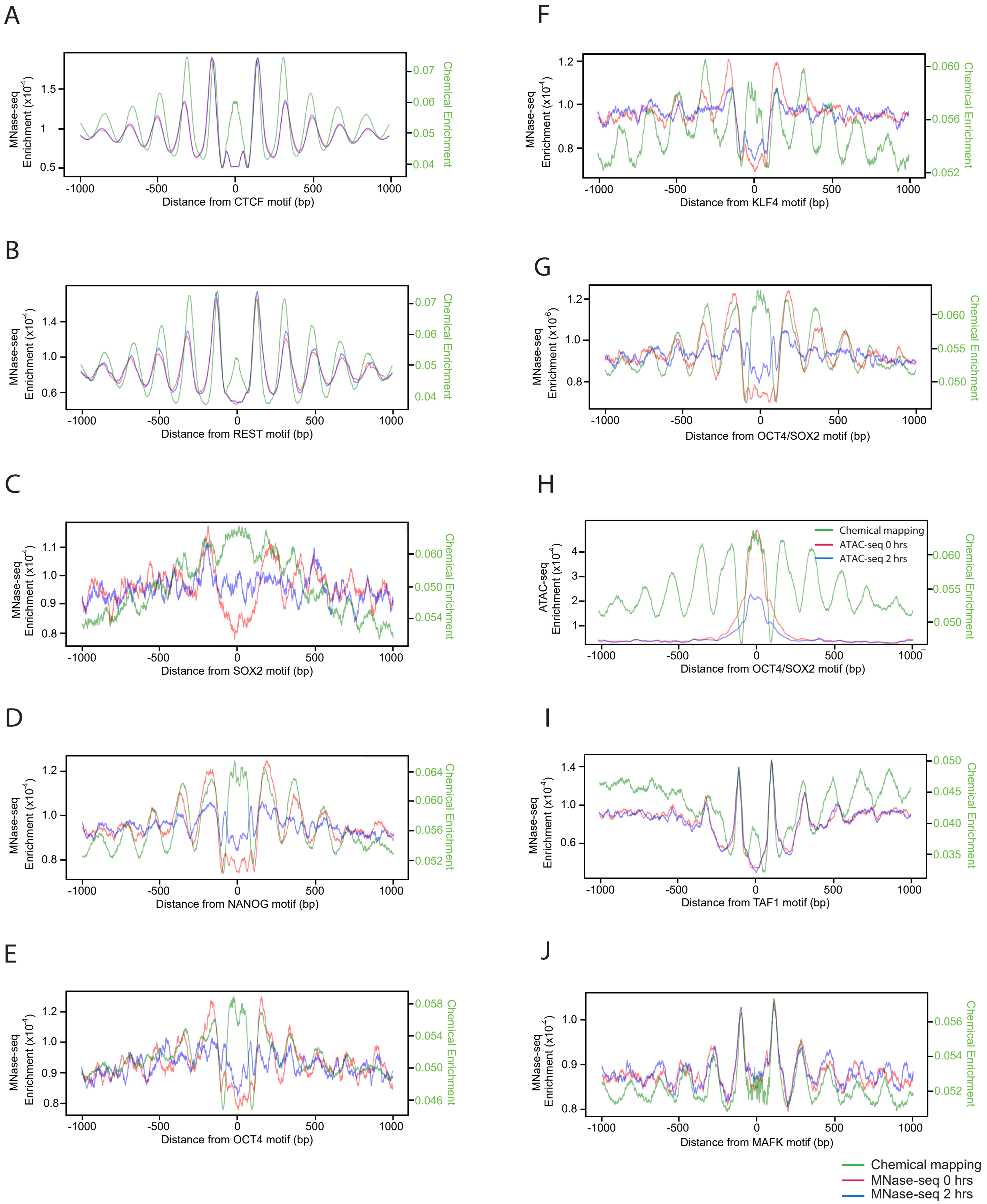

Figure S1 (related to Figure 3): The ARID1A-dependent nucleosome positioning adjacent to transcription factors. A-G, I-J) The distribution of MNase-seq and chem-seq signal adjacent to binding sites for the transcription factors indicated. Nucleosome locations as determined by MNase-seq are shown in red. MNase-seq following ARID1A degradation for 2 h in blue. Chem-seq data is shown in green. The signal at individual motifs for SOX2 and OCT4 is similar to that at a combined motif consistent with these factors frequently binding to adjacent sites. H) ATAC-seq at OCT4/SOX2 sites indicating that ATAC signal is localised to the central ARID1A sensitive nucleosome.

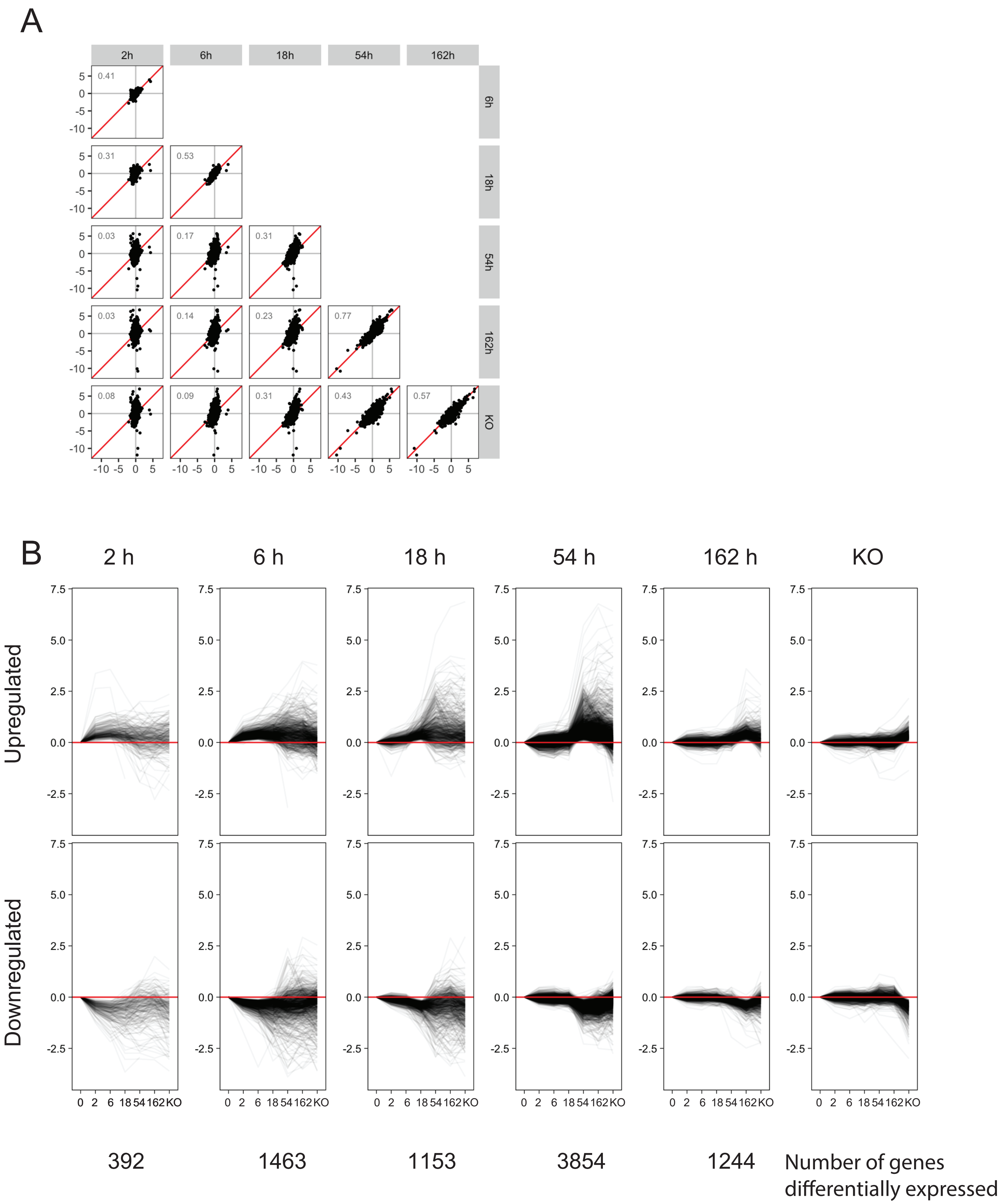

Figure S2 (related to Figure 4): Changes to the transcriptional landscape following loss of ARID1A.
A) Correlation between transcription of each gene at different time points. The transcriptome at early time points is distinct from that following growth for several days in the absence of ARID1A. However, over time the transcriptome adapts becoming progressively closer related to an ARID1A -/- line.
B) Transcriptional profiles of genes that are differentially expressed (FDR<0.05) at each time point. Each line represents the log2-fold change in expression of an individual gene across the time course.

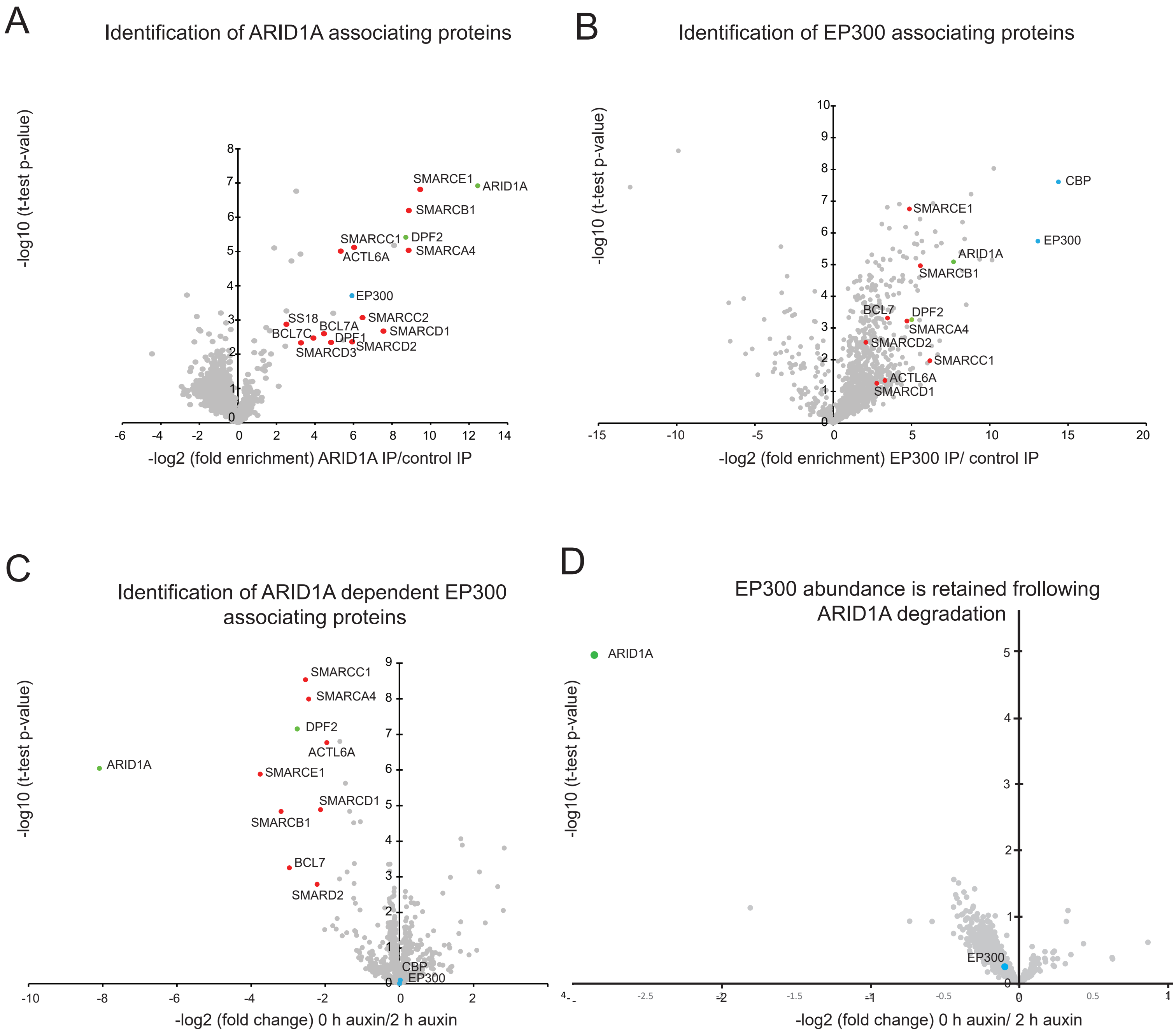

Figure S3 (related to Figure 5): Association of EP300 with SWI/SNF complexes is ARID1A dependent. A) ARID1A containing complexes were immunopurified and associating proteins identified by mass spectrometry. Many previously identified components of BAF complexes (red) and EP300 (blue) associate with ARID1A. The enrichment of each factor compared to an IP with a non-specific antibody is plotted against the p-value for the enrichment. B) Immunopurification of EP300 associated proteins followed by mass spectrometry results in enrichment for components of BAF complexes including core subunits (red) and BAF specific subunits (green). C) Following degradation of ARID1A association of SWI/SNF complex components (red) with EP300 is reduced. The fold change in enrichment of EP300 associated proteins prior to and 2 h following addition of auxin is shown. D) View of total cell proteomics dataset generated as in Figure 1C replotted to indicate that EP300 protein abundance is conserved following growth with auxin for 2 h.

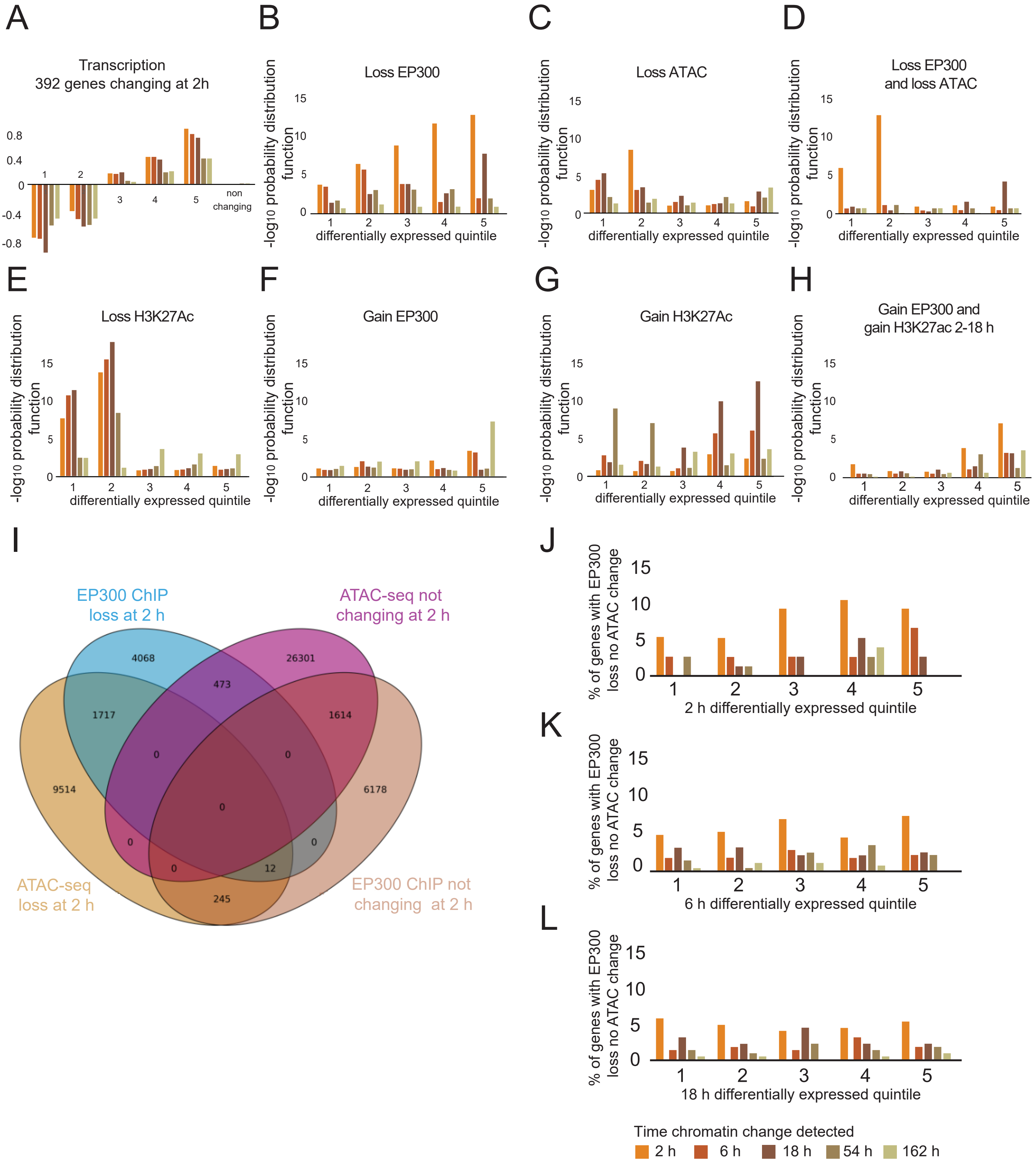

Figure S4 (related to Figure 5): Significance of enrichments at differentially expressed genes. By comparing the frequency of chromatin changes occurring at differentially expressed genes with the frequency of these changes at all genes the probability mass function can be calculated. This indicates the likelihood that the observed enrichment at differentially expressed genes would occur by chance. -Log10 probability mass functions are shown for different classes of chromatin change shown in Figure 5 G-M at genes differentially expressed after 2 h. For reference, A shows average fold-change in expression of each quintile of genes differentially expressed at 2 h (FDR<0.05) and is duplicated from Figure 5. B-H, show probability mass functions indicating the significance of intersects. Probability mass function is affected by changes in regions flanking all differentially expressed genes and needs to be considered in combination with the absolute enrichments shown in Figure 5. The sites of dual change

strongly linked to transcriptional up and down regulation have mass functions in the range 10^{-6} to 10^{-13} and are very unlikely to occur by chance. I) The intersect between ATAC-seq and EP300 ChIP at non changing sites and 2 h following degradation of ARID1A. 473 sites are detected where EP300 occupancy is reduced and ATAC signal is present but does not change following loss of ARID1A. Using the same format as Figure 5, the frequency with which up- and downregulated genes are associated with this class of change is shown in J. The changes are enriched for adjacent to upregulated genes. This may reflect EP300 acting as a co-repressor rather than a co-activator at a small subset of locations.

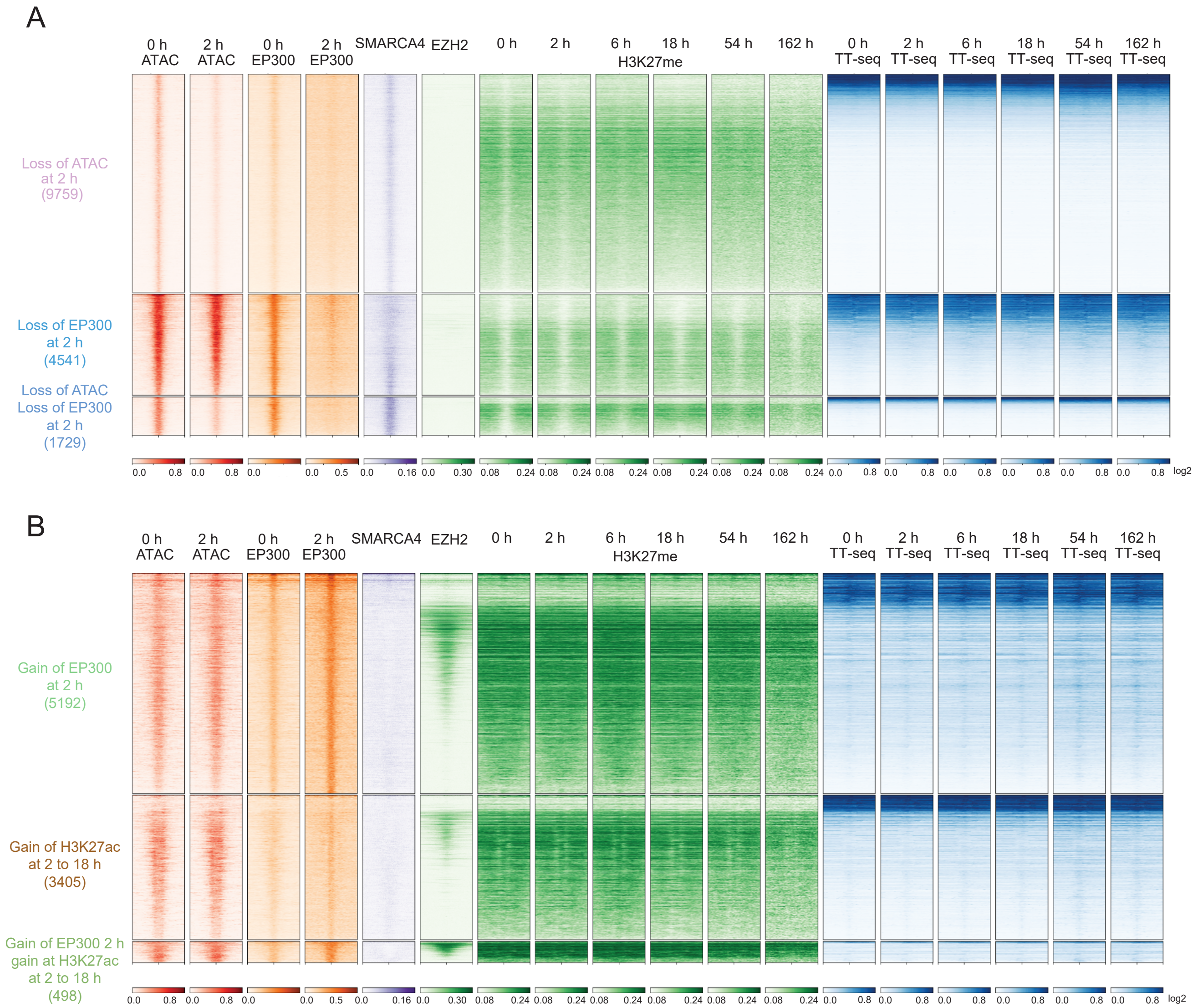

Figure S5 (related to Figure 5): Changes to Histone H3K27 tri-methylation and transcription at sites of ARID1A action. Groups of sites are selected as described in Figure 5 based on the intersecting peaks indicated in Figure 5B. Histone H3K27me3 ChIP and transcription (TT-seq) obtained at the indicated times following degradation of ARID1A are indicated. Heatmaps are centred with 2 kb either side.

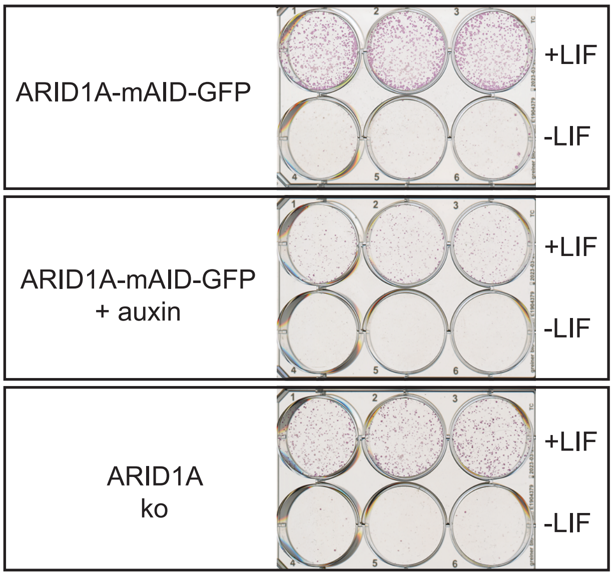

Figure S6 (related to Figure 7): ARID1A degron phenocopies pluripotency phenotypes observed in and ARID1A null line. ARID1A-mAID-GFP mES Cells, grown with and without 500 μM auxin, and ARID1A KO cells were grown as colonies for 6 days in the presence or absence of LIF and stained with alkaline phosphatase. ARID1A depleted and ARID1A null cells form fewer pluripotent colonies in the presence of LIF and fewer differentiated colonies following LIF withdrawal. Similar effects are observed in an ARID1A KO line. This is consistent with previous observations indicating that ARID1A is both required for mouse development (Gao et al., 2008), reprogramming ES cells (Singhal et al., 2010) and the action of pluripotency factors (Alver et al., 2017; Ho et al., 2009; King and Klose, 2017; Zhang et al., 2014) .
